# The serotonin receptor 2b (5-HT_2B_) modulates heart remodeling following myocardial infarction via regulation of Hippo pathway

**DOI:** 10.1016/j.isci.2026.114825

**Published:** 2026-01-27

**Authors:** Ryan Potter, Min Zi, Sukhpal Prehar, Tara McNulty, Dowan Kwon, Ellie England, Maram Almutairi, Nicholas Stafford, Ardiansah Bayu Nugroho, Efta Triastuti, Luc Maroteaux, Elizabeth J. Cartwright, Delvac Oceandy

**Affiliations:** 1 Division of Cardiovascular Sciences, Faculty of Biology, Medicine and Health, The University of Manchester, Manchester Academic Health Science Centre, Manchester, UK; 2 Division of Diabetes, Endocrinology and Gastroenterology, Faculty of Biology, Medicine and Health, The University of Manchester, Manchester Academic Health Science Centre, Manchester, UK; 3 Institut du Fer à Moulin, U1270 INSERM, Sorbonne Université, 17 rue du Fer à Moulin, 75005 Paris, France

**Keywords:** Biochemical mechanism, Human metabolism, Molecular network

## Abstract

Myocardial infarction (MI) is a leading cause of death globally. Following MI, the heart undergoes remodeling leading to heart failure. The Hippo pathway is a major regulator of cell growth and survival in cardiomyocytes. Here, we show that serotonin receptor 2B (5-HT_2B_) regulates the Hippo pathway in cardiomyocytes and modulates heart remodeling following MI. 5-HT_2B_ expression significantly enhanced the Hippo pathway effector Yes-associated protein (YAP) activity resulting in increased cardiomyocyte proliferation and decreased apoptosis. However, transgenic mice overexpressing 5-HT_2B_ in cardiomyocytes had a lower survival rate post-MI. Conversely, modified mRNA (modRNA)-mediated transient 5-HT_2B_ expression in the heart was sufficient to inhibit post-MI remodeling. Pharmacological screening of serotonergic compounds identified SB204741 as a modulator of the Hippo/YAP pathway in cardiomyocytes. SB204741 has been shown to protect the heart from adverse remodeling post-MI. Our findings identify 5-HT_2B_ as a regulator of the Hippo pathway that can be targeted to improve cardiac phenotype following MI.

## Highlights

• Serotonin receptor 2B (5HT_2B_) regulates the Hippo pathway and activates YAP in cardiomyocytes
• 5HT_2B_ promotes cardiomyocyte proliferation and survival *in vitro*
• Transgenic overexpression of 5HT_2B_ exacerbates hypertrophic remodeling following MI
• modRNA delivery of exogenous 5HT_2B_ was partially protective against MI-induced remodeling

## Introduction

Heart attack or myocardial infarction (MI) is a leading cause of mortality in the global population.^1^ During an MI, abrupt occlusion of a coronary artery supplying the myocardium causes severe ischemia. This initiates massive necrosis and apoptosis which eventually results in cardiomyocyte loss.^2^ It is understood that the adult mammalian cardiomyocyte possesses a negligible capacity for self-renewal^3^^,^^4^; therefore, the injured heart is unable to substantially repair itself and hence it undergoes a series of complex and profound alterations in its structure and function, referred to as adverse cardiac remodeling, characterized by dilatation of the infarcted wall, deposition of interstitial fibrosis, and enlargement of surviving cardiomyocytes.^5^ Collectively, these changes lead to the progressive loss of cardiac function that deteriorates into chronic heart failure (HF).

In mammals, embryonic cardiomyocytes exhibit robust proliferative capacity during development and upon injury, while adult cardiomyocytes are considered to be terminally differentiated.^3^^,^^6^^,^^7^ Interestingly, advances in the understanding of cardiomyocyte cell-cycle control suggest that reactivating endogenous cardiomyocyte proliferation could become a viable approach to repair the adult mammalian heart post-MI.^8^ Among other signaling pathways involved in the regulation of cell proliferation, the highly conserved developmental Hippo pathway has been identified as a key endogenous regulator of cell proliferation and survival in cardiomyocytes, and there is accumulating evidence that modulating this pathway can improve cardiac function and survival following MI.^9^^,^^10^^,^^11^^,^^12^

The mammalian core components of the Hippo pathway consist of the sterile 20-like protein kinase (MST1/(2) and its adaptor protein Salvador (SAV1), which upon upstream activation phosphorylates large tumor suppressor (LATS1/(2) and the LATS1/2-interacting protein MOB1, which in turn leads to phosphorylation and cytoplasmic retention of the transcriptional coactivators, Yes-associated protein (YAP), and transcriptional coactivator with PDZ-binding motif (TAZ).^13^ Targeting the Hippo pathway to facilitate cardiac regeneration would aim at elevating YAP and TAZ activity, although direct pharmacological activation of YAP and TAZ is challenging as these proteins have no known catalytic activity.^14^ However, these effectors are regulated by an upstream complex interplay of intrinsic cell machineries such as cell-cell contact, cell polarity, and actin cytoskeleton dynamics, in addition to a wide range of extrinsic signals, including cellular energy status, mechanical cues, and hormonal signals mediated by G-protein-coupled receptors (GPCRs).^13^ GPCR regulation of the Hippo pathway is particularly intriguing as GPCRs are highly targetable for therapeutics, with some 35% of currently available commercial drugs targeting GPCRs.^15^ Therefore, identifying novel regulators of the Hippo pathway that can be targeted pharmacologically through GPCRs could be a promising translational approach to promote cardiac regeneration.

A genome-wide RNAi screen study has identified novel modulators of Hippo signaling in *Drosophila melanogaster*.^16^ Based on the available dataset (GenomeRNAi accession number GR00218-S), the *Drosophila* serotonin receptor (5-HT_7_) gene is shown to be associated with the regulation of the Hippo pathway. Interestingly, in a separate study, another serotonin receptor, 5-HT_1A_, has been identified as a possible negative Hippo pathway regulator in mammalian HEK293 cells.^17^ On the basis of these findings, we hypothesized that serotonin (5-HT) signaling in the cardiomyocytes may be associated with Hippo pathway and may become a target of interest for modulating adverse cardiac remodeling in pathological conditions.

Several lines of evidence suggest that serotonin signaling plays an essential role in cardiac development. For example, 5HT is detectable in the mouse and human heart alongside 5HT synthesizing enzymes and several types of 5HT receptor coupled to different G-proteins, notably 5-HT_2A_, 5-HT_2B_, and 5-HT_4_, both in the myocardium and nonmyocyte tissue.^18^ The importance of 5-HT_2B_ expression in several cell types in the heart has been described, for example, in fibroblasts and myofibroblasts, 5-HT_2B_ plays an important role during cardiac adverse remodeling,^19^ whereas in cardiomyocytes it is involved in modulating myocyte growth and development.^20^ The 5-HT_2B_ receptor, a GPCR coupled to Gα_q/11_, is among the most highly expressed 5HT receptors in the heart^21^ and has been shown to be critical for viability, as mice with cardiac-specific deletion of 5-HT_2B_ exhibit embryonic and neonatal lethality caused by cardiac defects including a lack of trabeculae formation and dilated cardiomyopathy.^20^^,^^22^ In contrast, mice with cardiac-specific overexpression of 5-HT_2B_ exhibit compensated ventricular hypertrophy, with thickened ventricular walls and increased cardiomyocyte cell number and cell size.^23^ Despite these profound effects on cardiac development, the mechanism through which 5-HT_2B_ exerts these effects remains unclear.

Therefore, in this study, we investigated whether serotonin signaling, in particular signaling regulated by the 5-HT_2B_ receptor, may regulate the Hippo pathway in cardiomyocytes and whether this can be targeted to control cardiac remodeling following ischemic stress.

## Results

### Serotonin receptor 2B (5-HT_2B_) regulates the hippo pathway in neonatal rat cardiomyocytes

**Figure 1:**
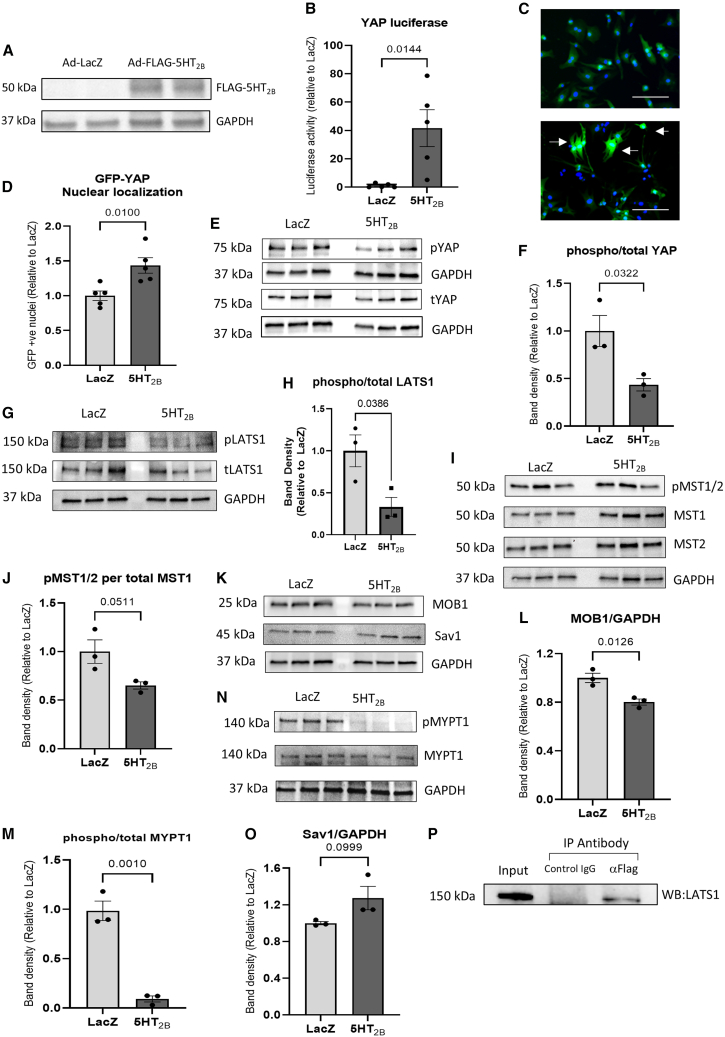
Regulation of the Hippo pathway by 5-HT_2B_ in cardiomyocytes (A) Western blot confirmation that Ad-FLAG-5-HT_2B_ infection in NRCMs resulted in the expression of FLAG- 5-HT_2B_ that was absent in Ad-LacZ-infected controls (representative image of *n* = 3 independent experiments). (B) YAP transcriptional activity was enhanced in NRCMs following 5-HT_2B_ overexpression (*n* = 5 independent experiments with five technical replications in each experiment). (C) Representative images of GFP-YAP nuclear translocation in NRCMs overexpressing 5-HT_2B_ or control LacZ (scale bars, 100 μm). (D) Quantification of NRCMs with nuclear GFP-YAP showed that cells overexpressing 5-HT_2B_ exhibited a higher proportion of nuclear YAP (*n* = 5 independent experiments with three technical replicates in each prep). (E and F) (E) Western blots and (F) quantification of band density showing a reduction in YAP phosphorylation in NRCMs overexpressing 5-HT_2B_ (*n* = 3 independent experiments). (G and H) (G) Western blots and (H) quantification of band density showing a significant reduction in LATS1 phosphorylation in NRCMs overexpressing 5-HT_2B_ (*n* = 3 independent experiments). (I and J) (I) Western blots and (J) quantification of band density showing no significant change in MST1 phosphorylation in NRCMs overexpressing 5-HT_2B_ (*n* = 3 independent experiments). (K–M) (K) Western blots and (L) quantification of band density showing a reduction in expression of the LATS1 adaptor protein MOB1 in NRCMs overexpressing 5-HT_2B_ (*n* = 3 independent experiments). (K) Western blots and (M) quantification of band density showing no change in expression of the MST adaptor protein Sav1 in NRCMs overexpressing 5-HT_2B_ (*n* = 3 independent experiments). (N and O) (N) Western blots and (O) quantification of MYPT1 phosphorylation in NRCMs overexpressing 5-HT_2B_ indicated a possible role for ROCK1 linking 5-HT_2B_ with the Hippo pathway (*n* = 3 independent experiments). (P) Immunoprecipitation analysis in cardiomyocytes overexpressing Flag-5-HT_2B_ using anti-Flag antibody followed by western blot detection using anti-LATS1 indicated possible interaction between 5-HT_2B_ and LATS1. All data are presented as mean ± SEM. Statistical tests used: (B, D, F, H, J, L, M, and O) Student’s *t* test.

We first investigated if 5-HT_2B_ regulates the Hippo pathway in isolated neonatal rat cardiomyocytes (NRCMs). Transduction of NRCMs with adenovirus expressing FLAG-5-HT_2B_ for 48 h was confirmed to induce the expression of FLAG-tagged protein corresponding to the molecular weight of 5-HT_2B_ (Figure 1A). We used a luciferase-based reporter to monitor YAP activity and found that 5-HT_2B_ overexpression induced a significant increase in YAP transcriptional activity compared to controls (Figure 1B). We then assessed YAP subcellular location using a GFP-YAP construct, and we observed a significantly higher proportion of nuclear YAP in cardiomyocytes overexpressing 5-HT_2B_ compared with controls, indicating increased activation of YAP following 5-HT_2B_ overexpression (Figures 1C and 1D). This was supported by western blot analysis of whole cell lysates which determined that YAP phosphorylation was significantly decreased (indicating that YAP activity was increased) in 5-HT_2B_ overexpressing NRCMs (Figures 1E and 1F). Western blot analyses of the core Hippo pathway components upstream of YAP also showed a significant decrease in LATS1 phosphorylation; however, no significant changes were detected in phosphorylation of MST1/2 (Figures 1G–1J). Consistently, we observed a reduced expression of the LATS adaptor protein MOB1 (Figures 1K–1L) but no difference in the expression of SAV1 (Figures 1K and 1M).

Previous studies have implicated Rho-associated coiled-coil kinase 1 (ROCK1) in mediating serotonin receptor activation.^24^ ROCK1 is also associated with LATS1 modulation.^25^ This prompted us to speculate that ROCK1 may be involved in 5-HT_2B_-induced LATS1 phosphorylation. To examine if 5-HT_2B_ regulates ROCK1 activity, we analyzed the phosphorylation of MYPT1, which is a key substrate of ROCK1.^26^ We found that 5-HT_2B_ overexpression led to a marked reduction in MYPT1 phosphorylation (Figures 1N–1O), indicative of reduced ROCK1 activity.

Furthermore, we conducted immunoprecipitation assay to investigate if 5-HT_2B_ interacts with LATS1. Results presented in Figure 1P show that LATS1 was co-precipitated with 5-HT_2B_ indicating a possible direct interaction between these proteins, supporting the notion that 5-HT_2B_ is associated with the Hippo pathway. We then used protein 3D structure and protein docking modeling tools to predict how 5-HT_2B_ and LATS1 might interact and form a complex. The model of the interaction is shown in Figure S1. Collectively, our data suggest that 5-HT_2B_ is a potent regulator of the Hippo pathway that activates YAP by dephosphorylating the upstream core kinase cassette in a mechanism that may involve signaling through ROCK1 and LATS1.

### 5-HT_2B_ overexpression promotes cardiomyocyte proliferation and survival *in vitro*

**Figure 2:**
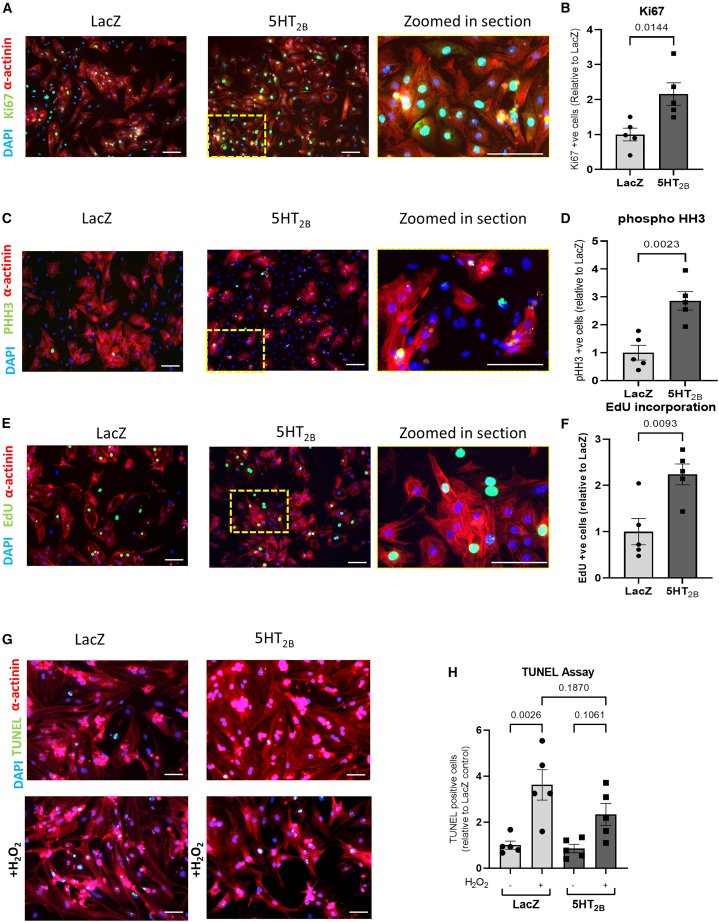
Effects of 5-HT_2B_ overexpression on cardiomyocytes *in vitro* (A and B) (A) Representative immunofluorescence images detecting the proliferative marker Ki67 (scale bars, 100 μm) and (B) quantification of Ki67-positive cardiomyocyte nuclei suggests 5-HT_2B_ overexpression increased proliferation (*n* = 5 independent experiments with three technical replicates in each experiment). (C and D) (C) Representative immunofluorescence images detecting the mitosis marker PHH3 (scale bars, 100 μm) and (D) quantification of PHH3-positive cardiomyocyte nuclei suggests 5-HT_2B_ overexpression increased mitosis events (*n* = 5 independent experiments with three technical replicates in each experiment). (E and F) (E) Representative immunofluorescence images detecting the thymidine analog EdU (scale bars, 100 μm) and (F) quantification of EdU-positive cardiomyocyte nuclei suggests 5-HT_2B_ overexpression increased EdU incorporation and therefore increased DNA replication (*n* = 5 independent experiments with three technical replicates in each experiment). (G and H) (G) Representative immunofluorescence images detecting TUNEL-labelled DNA breaks (scale bars, 50 μm), and (H) quantification of TUNEL-positive cardiomyocyte nuclei suggests that NRCMs overexpressing 5-HT_2B_ were more resistant to H_2_O_2_-induced apoptosis than controls (*n* = 5 independent experiments with three technical replicates in each experiment). All data are presented as mean ± SEM. Statistical tests used: (B, D, and F) Student’s *t* test; (H) one-way analysis of variance followed by *post hoc* test for multiple pairwise comparisons.

Our findings that 5-HT_2B_ overexpression inhibited the Hippo pathway and promoted YAP activation prompted us to investigate the effects of 5-HT_2B_ overexpression on cardiomyocyte proliferation and apoptosis in isolated NRCMs, given the key roles attributed to this pathway in regulating cardiomyocyte proliferation and apoptosis during development. Interestingly, immunofluorescence analyses revealed that 5-HT_2B_ overexpression significantly increased detection of the cell cycle marker Ki67 (Figures 2A and 2B) and the mitosis marker phospho-histone H3 (PHH3) in cardiomyocyte nuclei (Figures 2C and 2D), while an EdU incorporation assay detecting DNA synthesis identified an increase in EdU-positive cardiomyocytes following 5-HT_2B_ overexpression (Figures 2E and 2F).

To investigate the effects of 5-HT_2B_ overexpression on apoptosis, we treated NRCMs with 100 μM H_2_O_2_ for 2 h to mimic conditions of oxidative stress in the infarcted heart and conducted a TUNEL assay to detect apoptosis. H_2_O_2_ treatment significantly increased the percentage of apoptotic cardiomyocytes in the LacZ control group as expected, but not in the 5-HT_2B_ group (Figures 2G and 2H). Together, these data suggest that 5-HT_2B_ overexpression promotes cardiomyocyte proliferation and inhibits apoptosis *in vitro*.

### 5-HT_2B_ overexpression in human iPSC-derived cardiomyocytes increases YAP activity and proliferation

**Figure 3:**
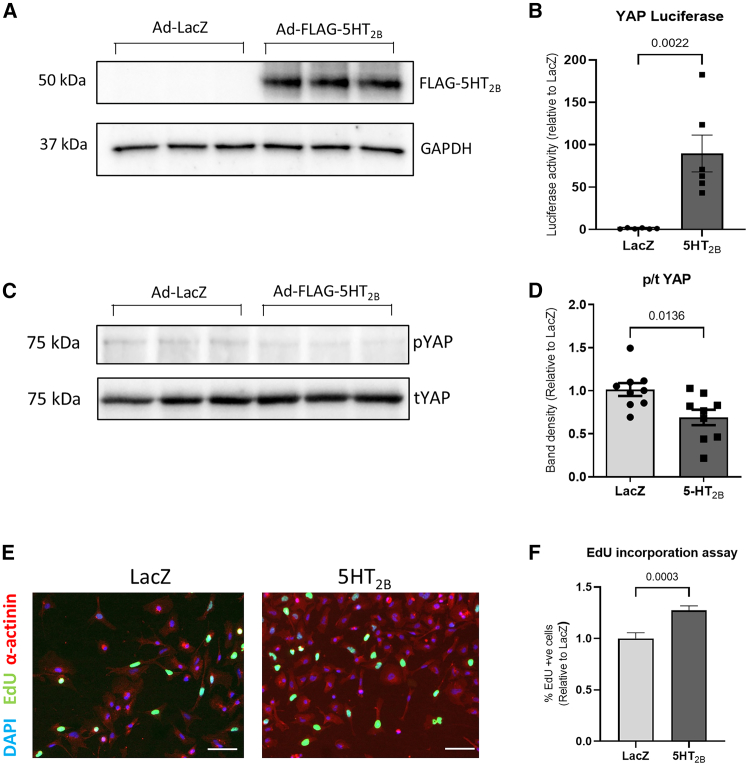
5-HT_2B_ regulates the Hippo pathway in human iPSC-CMs (A) Western blot confirmation that Ad-FLAG-5-HT_2B_ infection in hiPSC-CMs resulted in expression of FLAG-5-HT_2B_ that was absent in Ad-LacZ infected controls (*n* = 3 independent experiments). (B) YAP transcriptional activity was enhanced in hiPSC-CMs following 5-HT_2B_ overexpression (*n* = 6 independent experiments). (C and D) (C) Western blots and (D) quantification of band density showing a reduction in YAP phosphorylation in hiPSC-CMs overexpressing 5-HT_2B_ (*n* = 9 independent experiments). (E and F) (E) Representative immunofluorescence images detecting the thymidine analog EdU (scale bars, 50 μm) and (F) quantification of EdU-positive cardiomyocyte nuclei suggests 5-HT_2B_ overexpression increased EdU incorporation and therefore increased DNA replication (*n* = 3 independent experiments). Data are presented as mean ± SEM. Statistical tests used: (B, D, and F) Student’s *t* test*.*

To examine if 5-HT_2B_ overexpression modulates the Hippo/YAP pathway and induces proliferation in human cardiomyocytes, we transduced human iPSC-derived cardiomyocytes (iPSC-CMs) with adenovirus expressing 5-HT_2B_ (Figure 3A), replicated the effects of increased YAP activity as determined by a luciferase reporter (Figure 3B) and western blot (Figures 3C and 3D), and increased proliferation as determined by an EdU incorporation assay (Figures 3E and 3F), indicating that the role of 5-HT_2B_ is preserved in human cardiomyocytes.

### Effects of transgenic overexpression of 5-HT_2B_ on cardiac function and remodeling following myocardial infarction

**Figure 4:**
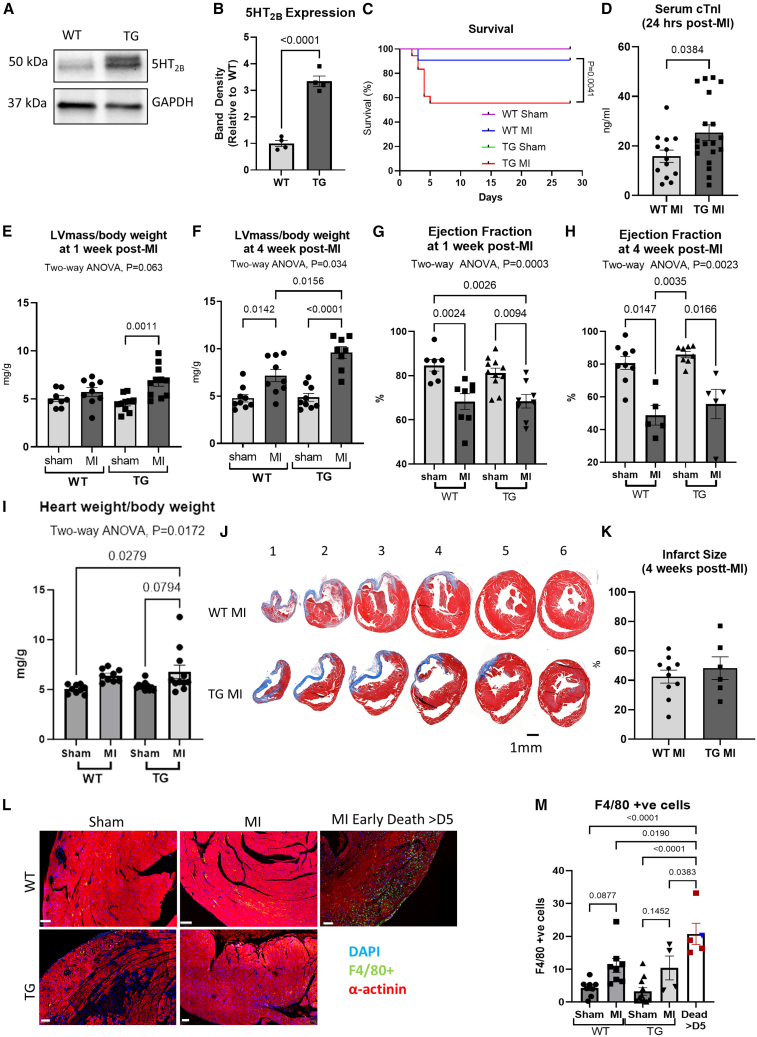
Effects of transgenic overexpression of 5-HT_2B_ on cardiac function and remodeling 4 weeks following myocardial infarction (A and B) (A) Western blot analysis of protein lysates from 12-week-old 5-HT_2B_^cTG^ (TG) and WT mice and (B) quantification of 5-HT_2B_ overexpression (*n* = 4 in each group). (C) TG mice survival to 4 weeks was markedly lower than that of WT mice subjected to MI (WT-sham, *n* = 9; WT-MI, *n* = 11; TG-sham, *n* = 11; TG-MI, *n* = 18 at the beginning of experiments). (D) Serum cTnI titer 24 h post-MI was significantly elevated in TG mice compared to WT mice (WT-MI, *n* = 13; TG-MI, *n* = 19). (E) The LV mass/body weight ratio was increased 1 week post-MI in TG mice only (WT-sham, *n* = 8; WT-MI, *n* = 9; TG-sham, *n* = 10; TG-MI, *n* = 11). (F) The LV mass/body weight ratio was elevated in both WT and TG mice by 4 weeks post-MI and was significantly higher in TG mice (WT-sham, *n* = 9; WT-MI, *n* = 9; TG-sham, *n* = 9; TG-MI, *n* = 8). (G) Cardiac function as indicated by EF was decreased 1 week post-MI in TG mice only (WT-sham, *n* = 7; WT-MI, *n* = 8; TG-sham, *n* = 11; TG-MI, *n* = 8). (H) By 4 weeks post-MI, EF was not significantly different between WT and TG mice (WT-sham, *n* = 9; WT-MI, *n* = 5; TG-sham, *n* = 8; TG-MI, *n* = 5). (I) Cardiac hypertrophy indicated by whole heart weight normalized to body weight was increased following MI in TG mice (WT-sham, *n* = 9; WT-MI, *n* = 9; TG-sham, *n* = 11; TG-MI, *n* = 11). (J and K) (J) Representative Masson’s Trichrome-stained heart sections (scale bar = 1 mm) and (K) quantification of infarct size suggests TG and WT mice developed similar sized infarcts following MI (WT-MI, *n* = 10; TG-MI, *n* = 6). (L) Representative images of staining using macrophage marker F4/80 in heart tissue sections of WT and TG mice following MI. (M) Quantification of the number of macrophage infiltration following MI.(WT-sham, *n* = 8; WT-MI, *n* = 8; TG-sham, *n* = 11; TG-MI, *n* = 4; dead mice, *n* = 5). Data are presented as mean ± SEM. Statistical tests used: (B, D, and K): Student’s *t* test; (E–I): two-way ANOVA followed by *post hoc* multiple comparisons. (M): one-way ANOVA followed by *post hoc* multiple comparisons.

Our data showing that 5-HT_2B_ overexpression improved cardiomyocyte proliferation and survival *in vitro* prompted us to hypothesize that overexpression of this receptor in the heart *in-vivo* would produce protective effects against pathological stimuli. We therefore studied transgenic mice with cardiomyocyte-specific overexpression of 5-HT_2B_ (5-HT_2B_^cTG^). Western blot analysis of whole heart lysates showed that the 5-HT_2B_^cTG^ mice exhibited ∼2-fold overexpression of 5-HT_2B_ compared to wild-type (WT) littermates (Figures 4A and 4B).

We subjected these mice to MI by permanent left anterior descending (LAD) coronary artery ligation and evaluated their response over 4 weeks. Surprisingly, we found that 5-HT_2B_ overexpression conferred a significant survival disadvantage following MI, with only 56% of 5-HT_2B_^cTG^ mice surviving to the end of the experiment compared to 91% of WT mice, with all deaths notably occurring in the initial 5 days following MI (Figure 4C). Interestingly, the analysis of serum cardiac troponin I (cTnI) at 24 h post-LAD ligation showed that cTnI titers were significantly higher in 5-HT_2B_^cTG^ mice compared to WT mice, indicating that 5-HT_2B_ overexpression was associated with more extensive cardiac damage following MI (Figure 4D).

Echocardiography analysis found that 5-HT_2B_^cTG^ mice showed an increased LV mass/body weight ratio 1 week post-MI that was absent in WT mice (Figure 4E). By 4 weeks post-MI, the LV mass/body weight ratio was increased in both 5-HT_2B_^cTG^ and WT mice, and this increase was significantly higher in the 5-HT_2B_^cTG^ group (Figure 4F). Ejection fraction (EF) at 1 week post-MI was apparently decreased in 5-HT_2B_^cTG^ mice; however, no significant difference in EF was detected between genotypes by 4 weeks post-MI (Figures 4G and 4H). Further analysis of the structural dimensions of the mouse heart is presented in Figure S2, including LV diameters (Figures S2A–S2D) and wall thickness (Figure S2E–S2H). Two-way ANOVA analysis showed a trend of higher increase of LV internal diameters and thickening of the interventricular septal wall in 5-HT_2B_^cTG^ mice, indicative of cardiac dilation and hypertrophy. This was supported by postmortem analysis of cardiac size showing heart weight normalized to body weight was significantly increased in 5-HT_2B_^cTG^ mice following MI, but not in WT mice (Figure 4I), indicating a greater degree of post-MI hypertrophy in 5-HT_2B_^cTG^ mice. While an increase in cell size was indeed detected by histological analysis following MI (Figures S3A and S3B), no differences were found between genotypes.

Assessment of Masson’s Trichrome-stained heart sections revealed that the infarct sizes of surviving animals at 4 weeks post-MI were comparable in 5-HT_2B_^cTG^ and WT mice (Figures 4J and 4K). However, immunofluorescent staining of F4/80+ macrophages showed enhanced macrophage infiltration following MI in both genotypes, which was particularly elevated in those animals that died in the first week following MI (Figures 4L–4M), suggesting that excessive inflammation might be contributed to severity of cardiac phenotype.

Furthermore, we found no evidence of enhanced proliferation or survival in 5-HT_2B_^cTG^ mice as measured by Ki67 detection (Figures S3C and S3D) or TUNEL staining (Figures S3E and S3F), respectively. Overall, our data suggest that transgenic overexpression of 5-HT_2B_ in cardiomyocytes does not replicate the beneficial *in vitro* effects of 5-HT_2B_ overexpression with regard to proliferation and apoptosis but is detrimental to survival and associated with exacerbated hypertrophy and dilation following MI.

### Effects of modRNA-mediated transient expression of *Htr2b* on cardiac function and remodeling following myocardial infarction

In light of our data showing that acute 5-HT_2B_ overexpression promotes cardiomyocyte proliferation and survival *in vitro* while long term 5-HT_2B_ overexpression *in vitro* in 5-HT_2B_^cTG^ mice is detrimental to survival and associated with exacerbated hypertrophy post-MI, we reasoned that transient expression of the 5-HT_2B_ receptor may be a more suitable approach to promote cardiac regeneration *in vitro*. It is understood that *in vivo* transient expression of a gene in the heart can be achieved using modified mRNA (modRNA).^27^ Therefore, we conducted experiments to test the effects of transient expression of 5-HT_2B_ receptor using modRNA in the mouse heart following MI.

**Figure 5:**
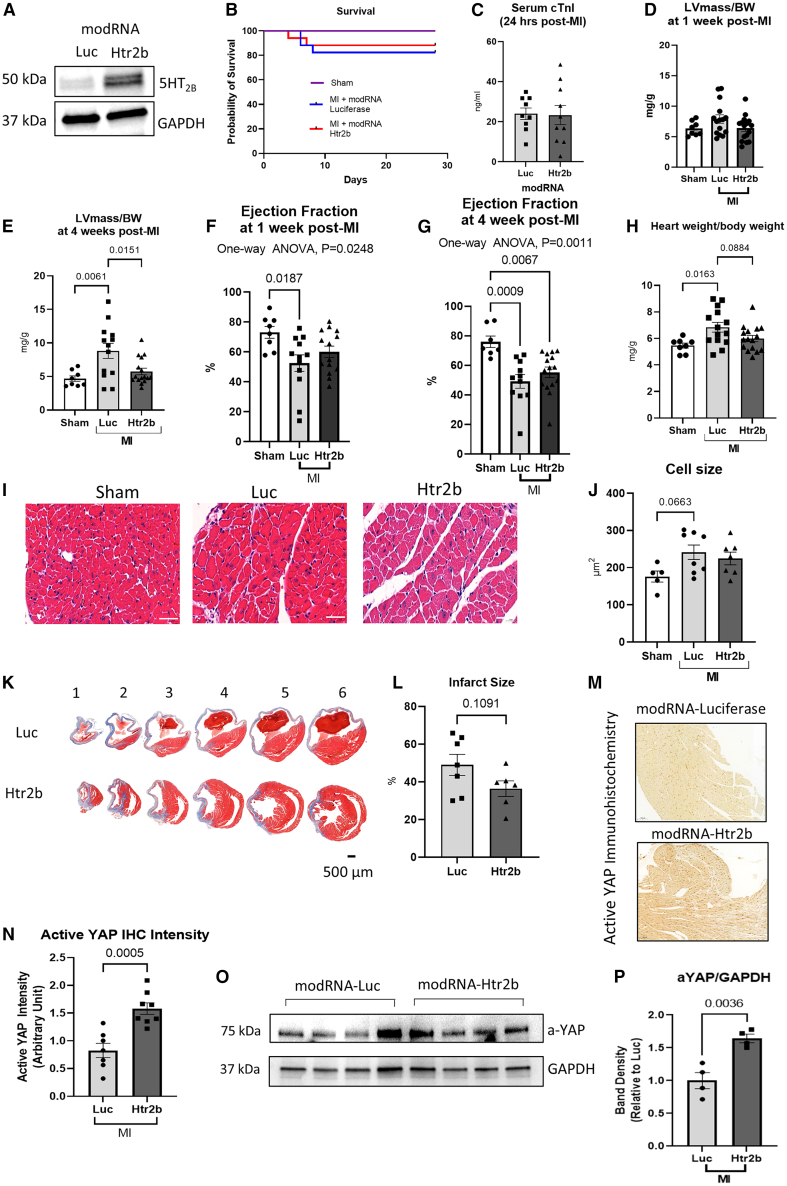
Effects of modRNA-mediated overexpression of 5-HT_2B_ on cardiac function and remodeling 4 weeks following myocardial infarction (A) Initial analysis was performed to validate modRNA-mediated overexpression of Htr2b. Wild-type mice were injected with either modRNA-Htr2b or modRNA-luciferase intracardially (*n* = 1 each). Western blot analysis showed expression of 5-HT_2B_ protein in mouse injected with modRNA-Htr2b. (B) Overall survival of mice did not significantly differ between modRNA groups at 4 weeks post MI (sham, *n* = 8; modRNA-Luc, *n* = 17; modRNA-Htr2b, *n* = 17 at the beginning of experiment). (C) Serum cTnI titer 24 h post-MI was similar in both modRNA groups (modRNA-Luc, *n* = 9; modRNA-Htr2b, *n* = 10). (D) The LV mass/body weight ratio was unchanged 1 week post-MI (sham, *n* = 8; modRNA-Luc, *n* = 15; modRNA-Htr2b, *n* = 16). (E) The LV mass/body weight ratio was significantly higher 4 weeks post-MI in modRNA-Luc mice but not in modRNA-Htr2b mice (sham, *n* = 8; modRNA-Luc, *n* = 13; modRNA-Htr2b, *n* = 16). (F–G) Cardiac function indicated by EF was similar at 1 week (sham, *n* = 8; modRNA-Luc, *n* = 12; modRNA-Htr2b, *n* = 14) and 4 weeks (sham, *n* = 7; modRNA-Luc, *n* = 11; modRNA-Htr2b, *n* = 15) post-MI in both modRNA groups. (H) Cardiac hypertrophy indicated by whole heart weight normalized to body weight was increased following MI in the modRNA-Luc mice but was unchanged in modRNA-Htr2b mice (sham, *n* = 8; modRNA-Luc, *n* = 14; modRNA-Htr2b, *n* = 16). (I and J) (I) Representative histological heart sections (scale bars, 40 μm) and (J) quantification of cell size indicated that relative to sham mice cell size appeared to increase more in the modRNA-Luc group (sham, *n* = 5; modRNA-Luc, *n* = 8; modRNA-Htr2b, *n* = 7). (K and L) (K) Representative Masson’s trichrome-stained heart sections (scale bars, 500 μm), and (L) quantification of infarct size showed no significant changes were elicited by modRNA with respect to infarct size (modRNA-Luc, *n* = 7; modRNA-Htr2b, *n* = 6). (M) Immunohistochemistry (IHC) analysis of active YAP expression on cardiac tissue sections of mice treated with modRNA-luciferase or modRNA-Htr2b following MI (scale bar, 100 μm, representative images of modRNA-Luc, *n* = 7; modRNA-Htr2b, *n* = 8). (N) Quantification of IHC signal suggests a significantly higher level of active YAP in heart sections treated with modRNA-Htr2b (modRNA-Luc, *n* = 7; modRNA-Htr2b, *n* = 8). (O) Western blots for the detection of active-YAP and GAPDH expressions in heart tissues treated with modRNA-Htr2b or modRNA-luciferase (representative image of modRNA-Luc, *n* = 4; modRNA-Htr2b, *n* = 4). (P) Quantification of band density showed a significant increase in active-YAP level in heart tissues following injection with modRNA-Htr2b (modRNA-Luc, *n* = 4; modRNA-Htr2b, *n* = 4). Data are presented as mean ± SEM. Statistical tests used: (C, L, N, and P): Student’s *t* test; (D–H and J): one-way ANOVA followed by *post hoc* multiple comparisons.

We first validated our modRNA constructs in H9C2 cells (Figures S4A–S4C) before proceeding to animal experiments. Western blot analysis of whole heart lysates collected 48 h following injection confirmed that modRNA-Htr2b upregulated expression of 5-HT_2B_ by > 2-fold (Figure 5A) while an *in vivo* imaging system confirmed that modRNA-Luc produced a strong bioluminescent signal localized to the heart (Figure S4D).

We then induced MI in 12-week-old WT C57BL/6J mice by permanent ligation of the LAD, followed immediately by intracardiac injection of 50 μg of modRNA-Htr2b or control luciferase (modRNA-Luc), and maintained the mice for 4 weeks. In contrast to the transgenic model, modRNA-Htr2b injection did not reduce the overall survival rate of mice compared to controls (Figure 5B). There was no significant difference in serum cTnI titers between the modRNA-Htr2b and the modRNA-Luc groups, indicating a comparable extent of cardiac damage following MI (Figure 5C).

We performed echocardiography analysis to assess cardiac morphology and function. We found that the LV mass/body weight ratio was beginning to increase 1 week post-MI in the modRNA-Luc group only, and by 4 weeks post-MI the LV mass/body weight ratio was significantly higher in the modRNA-Luc mice compared to controls but was unchanged in the modRNA-Htr2b group (Figures 5D and 5E), indicating a possible protection against hypertrophic remodeling in the modRNA-Htr2b group.

There was no significant change to EF at either 1 week or 4 weeks post-MI (Figures 5F and 5G). Further analysis of the structural dimensions of the mouse heart detected no significant changes 1 week post-MI; however, by 4 weeks post-MI, the modRNA-Luc mice exhibited significantly increased LV internal diameters and thickening of the interventricular septal and posterior walls, which were unchanged in the modRNA-Htr2b mice (Figures S5A–S5H), suggesting that modRNA-Htr2b was cardioprotective against dilation and hypertrophy following MI. This was supported by postmortem analysis of cardiac size showing heart weight normalized to body weight was significantly elevated only in the modRNA-Luc mice (Figure 5H) and not in the modRNA-Htr2b mice.

In keeping with the echocardiography data, histological analysis showed that relative to sham mice, cell size appeared to increase more in the modRNA-Luc mice than in the modRNA-Htr2b mice (Figures 5I and 5J), although differences were modest and did not reach significance. Analysis of Masson’s Trichrome-stained heart sections after MI demonstrated that infarct sizes were not significantly different between the modRNA groups (Figures 5K–5L).

Immunofluorescence analysis showed no significant elevation in apoptosis detected by TUNEL staining, which was negligible in all groups (Figures S6A and S6B), or in proliferation detected by Ki67 staining, although this appeared to trend highest in the modRNA-Htr2b group (Figures S6C and S6D). However, the analysis of active YAP level in cardiac sections demonstrated a significantly higher active YAP in mice injected with modRNA-Htr2b (Figures 5M–5P), indicating a possible modulation of the Hippo/YAP pathway. Collectively, these data suggest that transient expression of 5-HT_2B_ in the heart mediated by modRNA-Htr2b may improve the cardiac phenotype post-MI by attenuating hypertrophy and dilatation.

### Serotonergic screening for YAP-activating compounds

To further interrogate our hypothesis that 5-HT signaling in the heart may be a target of interest for controlling cardiac remodeling via the Hippo pathway, and to address the opposing effects we observed in our transgenic and modRNA mouse models, we conducted a screening analysis of serotonergic compounds aiming to identify YAP-activating drugs using a luciferase reporter system.

**Figure 6:**
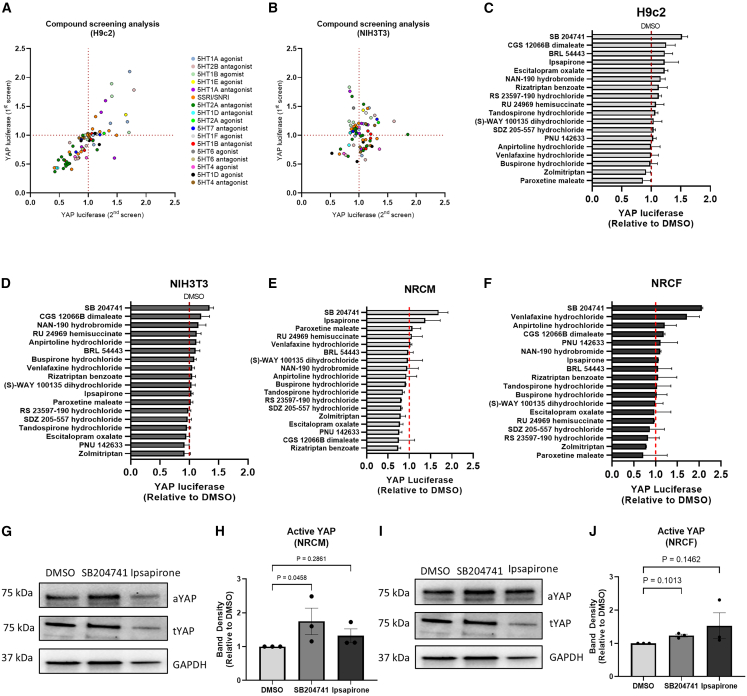
Serotonergic screening for YAP-activating compounds (A and B) (A) First-round screening data identified possible YAP modulating compounds in the H9C2 cardiomyoblast cell line (*n* = 3 independent experiments) and (B) the NIH/3T3 fibroblast cell line (*n* = 3 independent experiments). (C and D) 18 lead compounds were selected on the basis of differential YAP activity in H9C2 cells compared with NIH3T3cells. (E and F) Second-round screening data in isolated primary NRCMs and primary NRCFs (*n* = 3 independent experiments). (G–J) (G) Western blot and (H) quantification of active YAP expression in primary cardiomyocytes (NRCMs) treated with 1 μM SB204741 or 1 μM ipsapirone for 24 h (*n* = 3 independent experiments), and (I) western blot and (J) quantification of active YAP expression in primary fibroblasts (NRCFs) treated with 1 μM SB204741 or 1 μM ipsapirone for 24 h (*n* = 3 independent experiments) suggests that SB204741 modulates the Hippo pathway in cardiomyocytes but not in cardiac fibroblasts. Data are presented as mean ± SEM. Statistical tests used: (C–F) one-way analysis of variance followed by *post hoc* test for multiple pairwise comparisons. (H and J) Kruskal-Wallis test followed by *post hoc* multiple comparisons.

We first performed screening analysis using cardiac myoblast (H9c2) and fibroblast (NIH3T3) cell lines. We screened 110 known serotonergic compounds to identify drugs that activate YAP (Figures 6A and 6B). From the screening analysis, we identified 18 lead compounds that modulated YAP activity in either cell line (Figures 6C and 6D). We then tested the 18 compounds on YAP activation in isolated primary cardiomyocytes and fibroblasts (Figures 6E and 6F), confirming differential YAP-activating capacities of serotonergic compounds in cardiomyocytes and fibroblasts. Interestingly, from these experiments, we identified the compound SB204741, a selective antagonist of the 5-HT_2B_ receptor, to induce YAP activity in cardiomyocytes and cardiofibroblasts (neonatal rat cardiac fibroblasts [NRCFs]). Western blot analysis showed a trend of increased level of active YAP in NRCM following SB204741 treatment (Figures 6G and 6H); while in NRCF, SB204741 significantly enhanced YAP activity (Figures 6I and 6J). SB204741 has previously been shown to improve outcomes after MI through limiting the fibrotic process of scar formation by acting as a regulator of myofibroblast proliferation; as such, 5-HT_2B_ blockade has previously been implicated in inhibition of proliferation in the heart.^19^

### Effects of ipsapirone injections on cardiac function and remodeling following ischemia reperfusion

Our 5-HT screen also identified the compound ipsapirone, a partial agonist of the 5-HT_1A_ receptor, to activate YAP in cardiomyocytes but not in fibroblasts (Figures 6E and 6F). We therefore investigated whether ipsapirone would recapitulate the beneficial effects of YAP activation associated with cardiomyocyte proliferation without eliciting the detrimental effects of YAP activation associated with fibroblast proliferation.

**Figure 7:**
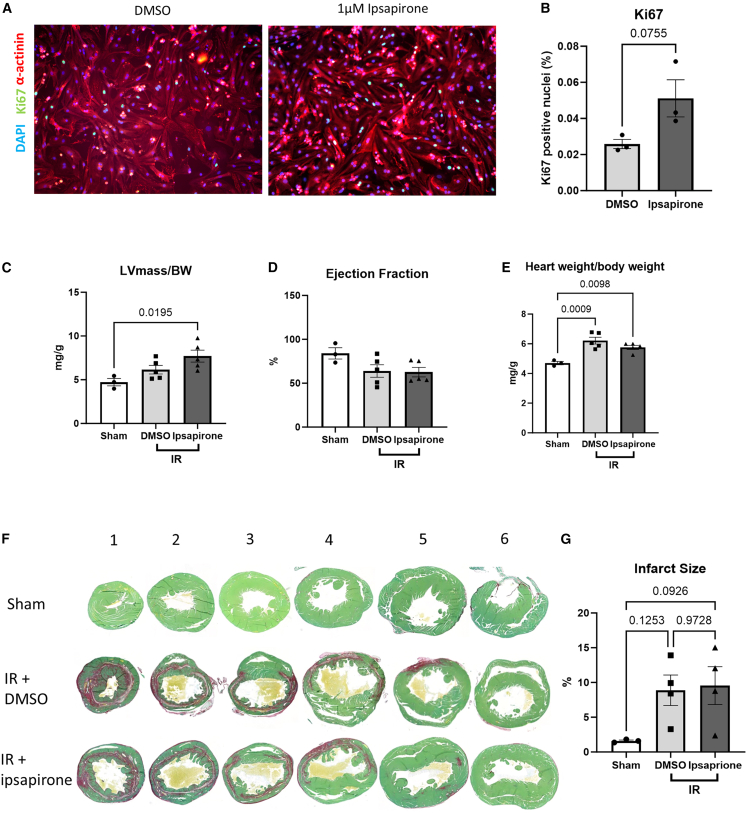
The effects of treatment with Ipsapirone in a model of IR (A and B) (A) Representative immunofluorescence images of Ki67-stained NRCMs (scale bars = 100 μm) and (B) quantification of Ki67 detection showed ipsapirone increased cell proliferation *in vitro* (*n* = 3 independent experiments). (C) The LV mass/body weight ratio was significantly increased 2 weeks post-IR in ipsapirone-treated mice only. (D) Cardiac function indicated by EF was unchanged 2 weeks post-IR. (E) Heart weight normalized to body weight was similarly elevated in both IR groups. (F and G) (F) Representative Picrosirius Red-stained heart sections (scale bar, 500 μm) and (G) quantification of infarct size suggests that ipsapirone did not affect infarct size compared to vehicle control (n number for C,D,E,G, sham, *n* = 3; IR-DMSO, *n* = 5; IR-Ipsapirone, *n* = 5). Data are presented as mean ± SEM. Statistical tests used: (B), Student’s *t* test*.* (C, D, E, and G) one-way ANOVA followed by *post hoc* test for multiple pairwise comparisons. Numbers above the bars indicate *p* values.

Contrary to SB204741, western blot analysis of whole cell lysates showed that the administration of 1 μM ipsapirone for 24 h did not significantly increase levels of active YAP in NRCMs (Figures 6G and 6H) and in NRCFs (Figures 6I and 6J). However, ipsapirone promoted cell proliferation as determined by increased Ki67 detection in NRCMs (Figures 7A and 7B).

We next conducted a pilot study to investigate the effects of ipsapirone in the context of IR using 12-week-old female WT C57BL/6J mice. IR with drug injections was chosen as a more translational model of MI compared to permanent LAD ligation; here, the LAD was temporarily ligated for 45 min prior to reperfusion and recovery and then 5 mg/mL ipsapirone or DMSO was delivered by daily intraperitoneal injections over 2 weeks. In our 2-week IR study, we found that ipsapirone injections were well-tolerated and did not affect mortality compared to DMSO injections or sham-operated mice, with just one post-operative death observed in each IR group (data not shown). Echocardiography analysis indicated that the LV mass/body weight ratio was significantly increased in the ipsapirone group but not in the DMSO controls (Figure 7C) while EF was maintained similarly in both groups (Figure 7D). Further analysis of the structural dimensions of the mouse heart detected no significant changes between groups by 2 weeks post-IR (Figures S7A–S7D). Postmortem analysis of cardiac size showed heart weight normalized to body weight was significantly elevated in both IR groups (Figure 7E). Finally, analysis of Picrosirius Red-stained heart sections after IR demonstrated that ipsapirone injections had no significant effect on infarct size compared to DMSO controls (Figures 7F and 7G).

## Discussion

The key finding of this study is that overexpression of 5-HT_2B_ in cardiomyocytes promotes cell proliferation and survival in a mechanism involving LATS1-dependent regulation of YAP. Our data suggest that overexpression of this receptor in the heart *in vivo* can either improve or exacerbate the cardiac phenotype post-MI, which is likely influenced by the duration of 5-HT_2B_ overexpression and the cell type-specific expression of the receptor.

We found that overexpression of 5-HT_2B_ led to increased activity of the key Hippo pathway effector YAP in both cultured NRCMs and in human iPSC-CMs. This was associated with reduced LATS1 phosphorylation in both cell models, consistent with the current understanding that inhibition of the core Hippo kinase cascade results in elevated YAP activity.^13^ 5-HT_2B_ is a G_q/11_-coupled GPCR and expression of a constitutively active Gα_q/11_ mutant has previously been reported to strongly decrease YAP phosphorylation.^28^ 5-HT_2B_ activation stimulates phospholipase C (PLC) to hydrolyze phosphatidylinositol 4,5-bisphosphate (PIP_2_) and generate the production of diacylglycerol (DAG) and inositol 1,4,5-triphosphate (IP_3_), which subsequently enhances PKC signaling and elevates intracellular Ca^2+^ concentration, respectively.^29^ 5-HT_2B_ has also been reported to stimulate Src, Fyn, and Yes family kinases,^30^ a finding that is relevant to our recent observation on the regulation of YAP by this receptor. However, we are among the first to identify a role for 5-HT_2B_ in modulating the Hippo pathway via LATS1 in cardiomyocytes. The 5-HT_2B_-dependent YAP regulation may involve ROCK1 signaling. Our finding that 5-HT_2B_ form protein interaction with LATS1 further strengthens the idea of the link between this receptor and the Hippo signaling pathway.

In colon cancer cells, serotonin has been shown to induce YAP activation via RhoA and ROCK1 signaling, which resulted in increased cell proliferation.^31^ In addition, ROCK1 has also been associated with LATS1 regulation.^25^ Thus, our data that 5-HT_2B_ overexpression led to a marked reduction in ROCK1 activity (as indicated by decreased phosphorylation of MYPT1) are in line with the idea that 5-HT_2B_ might modulate Hippo/YAP pathway via regulation of ROCK1.

Importantly, we observed that 5-HT_2B_ overexpression increased proliferation of both NRCMs and human iPSC-CMs. This is consistent with previous evidence showing that in the heart 5-HT_2B_ activation is mitogenic in valvular interstitial cells,^32^ while 5-HT_2B_ inhibition limits the proliferation and migration of myofibroblasts following MI.^19^ Moreover, transgenic mice overexpressing 5-HT_2B_ in cardiomyocytes exhibit ventricular hypertrophy associated with increased cell number and size,^23^ while cardiomyocyte-specific deletion of 5-HT_2B_ produces marked cardiomyopathy characterized by a loss of ventricular mass and an apparent reduction in cell number and size.^20^ Here, 5-HT_2B_ overexpression was associated with LATS1-dependent YAP activation and increased cardiomyocyte proliferation, consistent with accumulating evidence that the Hippo pathway is a key endogenous regulator of proliferation in the heart.^9^^,^^33^^,^^34^ Our data provide additional evidence of a pharmacologically targetable membrane receptor that modulates Hippo/YAP signaling in cardiomyocytes.

Another key finding of this study was that 5-HT_2B_ overexpression reduced apoptosis in NRCMs subjected to oxidative stress. 5-HT_2B_ activation has previously been shown to protect NRCMs against serum deprivation-induced apoptosis by limiting caspase-3 and caspase-9 cleavage downstream of cytochrome *c* release from the mitochondria. Mechanistically, 5-HT_2B_-dependent activation of ERK1/2 and PI3K/NF-KB signaling inhibited serum-deprivation induced upregulation of Bax and ANT1, respectively, to reduce mitochondrial membrane permeability and cytochrome *c* release.^35^ Interestingly, ROCK1 is a notable target of activated caspase-3 that has been reported to play an essential role in cardiomyocyte apoptosis,^36^ and our data suggest that ROCK1 activity was potently inhibited by 5-HT_2B_ overexpression, further implying that a cytoprotective effect is conferred by 5-HT_2B_. Here, we also associated the reduction in apoptosis with LATS1-dependent YAP activation in line with the reported pro-survival effects of elevated YAP activity, and although the relative contribution of YAP to improved NRCM survival is unclear, it is understood that the Hippo pathway cross-talks with both the MAPK/ERK and PI3K/Akt pathways previously implicated in 5-HT_2B_-mediated cytoprotection.^37^

Given the beneficial effects in NRCMs with respect to increased proliferation and survival following 5-HT_2B_ overexpression, the logical hypothesis was that overexpression of this receptor in cardiomyocytes *in vivo* would protect the heart against pathological damage following MI. However, our data showed that transgenic overexpression of 5-HT_2B_ in cardiomyocytes conferred a substantial survival disadvantage in response to MI, particularly during the acute phase of cardiac remodeling. Serum cTnI titers, which are proportional to the degree of tissue injury and disruption of myocyte membranes, were also significantly higher 24 h post-MI in 5-HT_2B_^cTG^ mice, indicating a larger myocardial damage in the transgenic mice at the initial phase after MI. In addition, while we did not detect a genotype-specific difference in F4/80+ macrophage infiltration in animals surviving to week 4 post-MI, we did observe significantly greater macrophage infiltration indicative of a more robust initial inflammatory response in those animals that died in the first week post-MI, the majority of which were 5-HT_2B_^cTG^ mice. This finding suggests that inflammation might contribute to the severity of cardiac phenotype and early death post-MI in 5-HT_2B_^cTG^ mice. It also indicates that 5-HT_2B_ and Hippo pathway might be associated with the regulation of cardiac inflammation. However, further studies are still needed to confirm this idea and to understand the mechanisms.

We also observed that cardiac hypertrophy and dilatation were exacerbated following MI in 5-HT_2B_^cTG^ mice. Previous studies have reported that cardiac hypertrophy and abnormal mitochondrial function occur in this mouse line under basal conditions.^23^ YAP has been implicated in mediating compensatory cardiac hypertrophy in response to pressure-overload through activation of the Warburg effect, thus our data linking 5HT_2B_ overexpression with enhanced YAP activity could explain the increased compensatory hypertrophy observed here through a similar mechanism.^38^ While hypertrophy is a necessary adaptive response to increased mechanical stress on surviving cardiomyocytes following MI, sustained and extensive hypertrophy is also associated with higher risk of mortality, arrhythmia, sudden cardiac death, and incidence of HF.^39^ It is therefore possible that MI-induced hypertrophy was exacerbated in 5-HT_2B_^cTG^ mice to increase mortality risk. However, the fact that all mouse deaths occurred between days 2 and 5 post-MI also gives rise to the possibility of other complications of MI in this group, such as left ventricular free-wall rupture or arrhythmia, as these complications most commonly occur within the first week after MI before adequate scar formation.^40^^,^^41^Overall, our data suggest that chronic induction of 5-HT_2B_ signaling starting from embryonic development might produce detrimental effects in the heart associated with greater inflammation and hypertrophy that were not present in our *in vitro* model. This is consistent with the notion that induction of pro-regenerative and pro-survival factors in the heart for extended durations may produce adverse effects, as has been shown recently in a large animal model.^42^

Following the results from the transgenic model, we then reasoned that acute and short-term expression of 5-HT_2B_ in the heart might result in different outcomes following MI. We utilized a modRNA approach for this purpose which is known to induce protein expression that peaks within 48 h and then degrades in ≤1 week.^27^ In contrast to our transgenic model, modRNA-Htr2b had no effect on the survival rate of mice following MI, and serum cTnI titers were similar between the modRNA-Htr2b and modRNA-Luc groups, indicating a comparable extent of cardiac damage at the beginning of MI. Moreover, modRNA-Htr2b appeared to be partially protective against MI-induced adverse remodeling as indicated by indices of cardiac hypertrophy and dilatation. However, we did not observe any significant differences in infarct size, cell proliferation, or apoptosis between the modRNA groups at the end of the 4-week experiment, although Ki67 detection trended highest in the modRNA-Htr2b group. It is worth noting that the modRNA approach taken here would be expected to induce 5-HT_2B_ expression in any cells that internalize the construct by passive dilution, including cardiac fibroblasts, endothelial cells, and cardiomyocytes^43^ and so we cannot exclude the contribution of non-myocyte 5-HT_2B_ expression in mediating the effects we observed in this study. Further experiments using a cardiomyocyte-specific modRNA expression system, for example, as described in an earlier study,^44^ are needed to examine the cell-specific effects of transient modRNA-mediated 5-HT_2B_ expression. While cardiomyocytes constitute the majority of cardiac cells by mass, other groups have shown that fibroblast-restricted 5-HT_2B_ inhibition is sufficient to modulate infarct expansion and cardiac function following MI.^19^ In addition, the phenotypes were observed at 4 weeks post-MI, when the effects of modRNA expression were diminished. Nevertheless, our data suggest that transient expression of 5-HT_2B_ in the heart was a better approach than long-term overexpression with respect to attenuating the post-MI cardiac phenotype.

Despite strong evidence showing that modulation of the Hippo/YAP pathway is beneficial in controlling adverse remodeling and preserving heart function post injury, targeting this pathway for therapeutic purposes remains elusive. This in part is due to the difficulties in finding pharmacological compounds that can modulate the core components of this pathway in a controlled manner.^45^ Our finding that a serotonin receptor can act as upstream regulator of this pathway will open a new avenue to target the Hippo/YAP pathway pharmacologically, since most of the serotonin receptors are well characterized^46^ and relatively large numbers of modulators have been identified. We therefore sought to identify modulators of serotonin receptors that can induce YAP activity in cardiomyocytes, but ideally not in other cell types. Our luciferase screen analysis showed that serotonergic compounds had differential YAP-activating capacities in cardiomyocytes and fibroblasts. Interestingly, we identified the compound SB204741 to activate YAP in cardiomyocytes. SB204741 is a known 5-HT_2B_ antagonist based on the contractile response of rat stomach tissue upon serotonergic stimulation.^47^ Thus, it is interesting that in terms of YAP activation in cardiomyocytes, SB204741 exerts similar effects to 5-HT_2B_ overexpression. Indeed, high-level overexpression of a receptor may produce different phenotypic outcomes from activating it using pharmacological compounds. It is possible that the exogenous protein interacts with other molecules and activates different signaling proteins. They may also exert a dominant negative effect by titrating the effectors. Further studies are needed to explain this finding.

Perhaps more importantly, studies by Snider and colleagues showed that SB204741 exhibited a strong protection against adverse remodeling post-MI in mice.^19^ In that study, SB204741 treatment improved cardiac function and remodeling following MI through limiting myofibroblast proliferation and infarct expansion.^19^ Although it is not described in the report, it is plausible that modulation of the Hippo/YAP pathway contributes to the improvement of the phenotype. Our screen analysis also indicated that ipsapirone, a partial 5HT_1A_ agonist, induced YAP-luciferase reporter activity in cardiomyocytes, and was able to induce proliferation of NRCMs. However, in our 2-week ischemia reperfusion (IR) study, despite inducing Ki67 positive cells, treatment with ipsapirone did not improve the infarct size and cardiac function after ischemic injury. It is possible that ipsapirone may induce YAP activity in other cell types, such as fibroblasts, which may reduce the beneficial effect of YAP activation in cardiomyocytes. However, further studies are still needed to understand these findings.

Nevertheless, our findings that the Hippo/YAP pathway can be modulated via GPCRs underline the interesting facts that modulation of GPCRs could produce beneficial effects in some cardiac pathological conditions. For example, activation of the Gq/11-coupled alpha-1A adrenergic receptor (α1A-AR) with the agonist dabuzalgron has been shown to protect the heart against doxorubicin-induced cytotoxicity by preserving mitochondrial function through an ERK1/2-dependent mechanism.^48^ Moreover, selective α1A-AR activation in cardiomyocytes protects against oxidative stress by improving fatty-acid dependent respiration, fatty acid oxidation, and electron transport chain enzyme activity in mitochondria.^49^

In conclusion, our study shows that 5-HT_2B_ is a novel and potent regulator of the Hippo/YAP pathway in cardiomyocytes that acts by inhibiting LATS1 kinase and enhancing YAP activity. However, long-term transgenic overexpression of 5-HT_2B_ in the heart exacerbated cardiac dilatation and hypertrophy and was detrimental to survival following MI, while transient modRNA-mediated expression of 5-HT_2B_ partially protected the heart against adverse cardiac remodeling post-MI. Screening of serotonergic compounds also identified SB204741 as a YAP modulator in cardiomyocytes and cardiac fibroblasts. Overall, this work identifies the 5-HT_2B_ receptor as a potential therapeutic target to reduce adverse cardiac remodeling post-MI.

### Limitations of the study

This study demonstrated that 5-HT_2B_ interacts with LATS1 and thereby regulates the Hippo pathway. However, the protein interaction was detected by a simple immunoprecipitation assay. Further analysis is needed to comprehensively determine which part of the proteins (the protein domains) is responsible for the interaction. Evaluation of protein interaction using different approaches (e.g., proximity ligation and FRET assays) is important to confirm the current finding.

Although we found that hypertrophy, and possibly inflammation, is exacerbated in 5-HT_2B_ cTG mice after MI, it will be important to confirm the cause of mortality in the transgenic mice following MI. Other possible causes of mortality during the first week of MI include arrhythmia and cardiac rupture. Future experiments can be conducted that (1) incorporate ECG measurements to assess any differences in electrical conduction and (2) investigate the changes of myocardial structure at the beginning of MI to provide experimental evidence regarding the cause of mortality.

The modRNA-based overexpression used in this study would induce 5-HT_2B_ expression in all types of cells, including cardiac fibroblasts, endothelial cells, and cardiomyocytes. It would be important to understand if cardiomyocyte-specific 5-HT_2B_ transient overexpression would produce more beneficial effects. This can be achieved by employing a cardiomyocyte-specific mod-RNA overexpression system.

## Resource availability

### Lead contact

Requests for further information, resources, and reagents should be directed to and will be fulfilled by the lead contact, Delvac Oceandy (delvac.oceandy@manchester.ac.uk).

### Materials availability

All unique/stable reagents generated in this study are available from the lead contact with a completed materials transfer agreement.

### Data and code availability

• All data reported in this paper will be shared by the lead contact upon request
• This paper does not report original code.
• Any additional information required to reanalyze the data reported in this paper is available from the lead contact upon request.

## Acknowledgments

This work was supported by 10.13039/501100000274British Heart Foundation (BHF) Program Grant RG/F/21/110055 to D.O. and 10.13039/501100000274BHF 4 years PhD studentship FS/17/67/33483B to E.J.C. to support R.P. We thank the University of Manchester Biological Services Facility and the Bioimaging Facilities for technical support.

## Author contributions

Study conception and design, D.O.; experimental design, R.P. and D.O.; *in vitro* experiments and data collection, R.P., T.M.N., D.K., E.E., M.A., E.T., and N.S.; *in vivo* experiments and data collection, R.P., M.Z., S.P., and A.B.N.; data analysis and interpretation, R.P. and D.O.; supervision of *in vivo* experiments, E.J.C.; provision of transgenic model, E.M.; and writing and editing the manuscript, R.P. and D.O.

## Declaration of interests

All authors declare they have no competing interests.

## STAR★Methods

### Key resources table

**Table:** 

REAGENT or RESOURCE	SOURCE	IDENTIFIER
**Antibodies**		

Anti-FLAG M2 Peroxidase	Cell Signalling Technologies	Cat. #8146;RRID:AB_10950495
5-HT_2B_	Bioorbyt	Cat. #orb352480
pMST1/2 (Thr183)/MST2 (Thr180)	Cell Signalling Technologies	Cat #49332; RRID:AB_2799355
MST1	Cell Signalling Technologies	Cat #3682; RRID:AB_2144632
MST2	Cell Signalling Technologies	Cat #3952; RRID:AB_2196471
Sav1	Cell Signalling Technologies	Cat #13301; RRID:AB_2798176
pLATS1 (Ser909)	Cell Signalling Technologies	Cat #9157; RRID:AB_2133515
tLATS1	Proteintech	Cat #17049-1-AP; RRID:AB_2281011
MOB1	Cell Signalling Technologies	Cat #13730; RRID:AB_2783010
pYAP (Ser127)	Cell Signalling Technologies	Cat #4911; RRID:AB_2218913
tYAP	SantaCruz	Cat #sc-376830; RRID:AB_2750899
aYAP	Abcam	Cat #ab205270; RRID:AB_2813833
pMYPT1 (Thr696)	Cell Signalling Technologies	Cat #5163; RRID:AB_10691830
MYPT1	Cell Signalling Technologies	Cat #2634; RRID:AB_915965
GAPDH-HRP	Cell Signalling Technologies	Cat #3683; RRID:AB_1642205
Vinculin	Abcam	Cat #ab91459; RRID:AB_2050446
Mouse anti-α-actinin	Sigma Aldrich	Cat #A7811; RRID:AB_476766
Rabbit anti-Ki67	Abcam	Cat #Ab15580; RRID:AB_443209
Rabbit anti-PHH3	Thermo Fisher Scientific	Cat #PA5-17869; RRID:AB_10984484
Anti-Mouse Alexa Fluor® 647	Jackson ImmunoResearch	Cat #115-605-072; RRID:AB_2338910
Anti-Mouse Alexa Fluor® 488	Jackson ImmunoResearch	Cat # 115-545-003; RRID:AB_2338840
Anti-Rabbit Alexa Fluor® 488	Jackson ImmunoResearch	Cat #711-545-152; RRID:AB_2313584
Anti-Rabbit Alexa Fluor® 488	Jackson ImmunoResearch	Cat #711-545-152; RRID:AB_2313584

**Bacterial and virus strains**		

DH5α competent *E.coli*	Thermo Fisher Scientific	Cat. #18265017
Ad-FLAG-5-HT_2B_	This paper	N/A
Ad-LacZ	Lestari B, et al. (2025). Nat Commun **14;16**:1650.	N/A
Ad-GAL4-TEAD	Triastuti E et al. (2019) Br J Pharmacol;**176**:3956-3971.	N/A
Ad-UAS-luciferase	Triastuti E et al. (2019) Br J Pharmacol;**176**:3956-3971.	N/A
Ad-GFP-YAP	Triastuti E et al. (2019) Br J Pharmacol;**176**:3956-3971.	N/A

**Biological samples**		

Plasmid pGAL4-TEAD	Addgene	Addgene plasmid # 24640
Plasmid pUAS-luciferase	Addgene	Addgene plasmid # 24343
Plasmid pGFP-YAP	Addgene	Addgene plasmid # 17843
pHtr2b	Origene	Cat. #MR224713
pGL3 luciferase reporter	Promega	Cat. #E1751

**Chemicals, peptides, and recombinant proteins**		

collagenase A	Roche	Cat. #05349907103
Pancreatin	Sigma-Aldrich	Cat. #P3292
bromodeoxyuridine (BrdU))	Sigma-Aldrich	Cat. #19-160
Laminin	Roche	Cat. #11243217001
paraformaldehyde (PFA)	Fisher	Cat. #10459113
4ʹ,6-diamidino-2-phenylindole (DAPI)	Thermo Fisher Scientific	Cat. #D1306
5-ethynyl-2′-deoxyuridine (EdU)	Thermo Fisher Scientific	Cat. #C10637
Antarctic phosphatase enzyme	New England Biolabs	Cat. #M0289
RNAiMAX	Thermo Fisher Scientific	Cat. #13778100
N1-methyl-pseudoUTP^∗^	Jena Bioscience	Cat. #NU890-L
Anti Reverse Cap Analog, 3'-O-Me-m7G(5')ppp(5')G (ARCA)^+^	Stratech	Cat. #B8175-APE

**Critical commercial assays**		

luciferase assay reagent	Promega	Cat. #E1483
Click-iT EdU Cell Proliferation Kit for Imaging	Thermo Fisher Scientific	Cat. #C10337
*In Situ* Cell Death Detection Kit	Roche	Cat. #11684795910
PureLink™ HiPure Plasmid Maxiprep Kit	Thermo Fisher Scientific	Cat. #K210007
MEGAscript® T7 Kit	Thermo Fisher Scientific	Cat. #AM1334
MEGAclear™ Transcription Clean-Up Kit	Thermo Fisher Scientific	Cat. #AM1908
(ELISA) CTNI-1-HSP kit	Life Diagnostics	N/A
Phusion Green Hot Start II High-Fidelity PCR Master Mix	Thermo Fisher Scientific	Cat. #F566S

**Experimental models: Cell lines**		

Human induced pluripotent stem cell line	Thermo Fisher Scientific	Episomal hiPSC line A18944
H9c2 rat cardiomyoblast	American Type Culture Collection (ATCC)	CRL-1446
NIH/3T3 mouse fibroblasts	American Type Culture Collection (ATCC)	CRL-1658

**Experimental models: Organisms/strains**		

αMHC-5-HT_2B_ Transgenic mice (129S2.CD1-Tg(Myh6-Htr2b)1Lum/Orl)	European Mouse Mutant Archive (EMMA)	EM:05938
C57BL/6J mice	Envigo	N/A
Sprague-Dawley rat	Charles River Laboratories	N/A

**Oligonucleotides**		

Genotyping fwd primer (5′-TGTAATCT TGATGAATGCAGTAGCC-3’	Merck	N/A
Genotyping reverse primer (5’ CAGAA GACATGTGATCACCTGATC-3’)	Merck	N/A

**Software and algorithms**		

GraphPad Prism v.10	GraphPad Software	N/A
ImageJ	NIH	N/A
ImageLab	Biorad	N/A
CaseViewer	3DHISTECH	N/A

### Experimental model and study participant details

#### Animal models

Animal studies were conducted in accordance with the United Kingdom Animals (Scientific Procedures) Act 1986 and approved by the University of Manchester Ethics Committee (PPL number: PP5982529). We investigated the role of 5-HT_2B_ in the heart following MI using 12-week-old male and female transgenic *129sv* mice with cardiomyocyte-specific overexpression of 5-HT_2B_ (5-HT_2B_^cTG^), alongside age-matched wild-type (WT) littermates. These mice were generated by fusing the cardiomyocyte-specific promoter α-myosin heavy chain (αMHC) to the entire coding sequence of the mouse 5-HT_2B_ gene (Htr2b) as previously described.^23^ We also investigated the role of 5-HT_2B_ in the heart following MI using 12-week-old wildtype male and female WT C57BL/6J mice (Envigo) injected with modified RNA constructs containing either the mouse Htr2b mRNA sequence or luciferase mRNA control. Mice were maintained in the University of Manchester Biological Services Facility under a 12 h light/dark cycle at 19–22 °C and 40–65% humidity with a standard chow diet.

#### Cell lines

The role of 5-HT_2B_ was characterised *in-vitro* using primary neonatal rat cardiomyocytes (NRCMs), human iPSC-derived cardiomyocytes (iPSC-CM) and the H9c2 rat cardiomyoblast cell line. H9C2 cardiomyoblasts and NIH/3T3 fibroblasts were from ATCC, maintained in culture and passaged at a ratio of 1:4 upon reaching 80% confluency. H9C2 and NIH/3T3 cells were used for 5-HT compound screening analysis and in validation experiments where described. Human iPSC-derived cardiomyocytes were also used to confirm the effects of 5-HT_2B_ overexpression in human cells. The human iPSC line used was the episomal hiPSC line A18944 supplied by Thermo Fisher Scientific. We did not perform cell authentication during this study. Cell lines were tested for mycoplasma contamination.

#### Primary cell cultures

Primary neonatal rat cardiomyocytes (NRCMs) isolated from 2- to 3-day-old Sprague-Dawley rats were used to test the effects of adenovirus-mediated overexpression of 5-HT_2B_ or control LacZ. Detailed methods for NRCM isolation, culture, and modulation of gene expression are written in the method sections below.

### Method details

#### Neonatal rat cardiomyocyte and fibroblast isolation and culture

To isolate NRCMs, 2–3-day old Sprague-Dawley rat pups (Charles River Laboratories) were terminated via cervical dislocation, immersed in 70% ethanol (Fisher) in water and decapitated prior to whole heart extraction by central chest incision. Excised hearts were immediately placed in ice-cold phosphate buffered saline (PBS; 137 mM NaCl, 2.7 mM KCl, 8 mM Na_2_HPO_4_ and 2 mM KH_2_PO_4_) (Sigma-Aldrich) and transferred to a class II biological safety cabinet. Epicardial fat was removed, hearts were cut longitudinally and transferred to 7 mL filter sterilised artificial digestive solution (ADS; 116 mM NaCl, 20 mM HEPES, 1 mM NaH_2_PO_4_, 5.5 mM glucose, 5.4 mM KCl, 0.83 mM MgSO_4_; pH 7.35) (Sigma-Aldrich) supplemented with 1.3 mg/mL collagenase A (Roche) and 6.25 mg/mL pancreatin (Roche) and placed in a shaking waterbath at 37°C for 5 min. The cell-containing ADS solution was collected and transferred through a cell strainer to a fresh bottle and 3 mL foetal bovine serum (FBS) (Gibco) was added to terminate enzymatic digestion. A further 7 mL ADS buffer was sequentially incubated with the hearts in this manner until all tissue was digested.

The pooled cell solution was centrifuged at 1200 rotations per min (rpm) for 5 min at room temperature (RT) and the supernatant was discarded. Cells were resuspended in pre-plating media (68% Dulbecco’s Modified Eagle Medium (DMEM), 17% Medium 199, 10% horse serum, 5% FBS, 2.5 mg/mL amphotericin B and 100 units/mL penicillin and streptomycin) (Gibco) and plated onto 40 mm tissue culture dishes (Corning) for 1 h in a 37°C/5% CO_2_ incubator to allow cardiac fibroblasts to adhere to the dish. The media containing the cardiomyocyte fraction was then carefully collected and total cell number was estimated using a Countess II automated cell counter (Thermo Fisher Scientific). Cells were then transferred to plating media (pre-plating media supplemented with 0.1% bromodeoxyuridine (BrdU)) (Sigma-Aldrich) and seeded onto either i) 9.6 cm^2^ 6-well plates (Corning) at a density of 2 x 10^6^ cells/well, or ii) 1.9 cm^2^ 24-well plates (Corning) at a density of 2.5 x 10^5^ cells/well and maintained in a 37°C/5% CO_2_ incubator. After 16 h, plating media was replaced with maintenance media (80% DMEM, 20% Medium 199, 1% FBS, 2.5 mg/mL amphotericin B, 100 units/mL penicillin and streptomycin and 0.1% BrdU) prior to commencing experiments. Furthermore, adherent cardiac fibroblasts (NRCFs) were transferred from the culture dishes to 6-well or 24-well plates after 24 h by trypsin-EDTA (Gibco) detachment and cultured in DMEM supplemented with 10% FBS and 100 units/mL penicillin and streptomycin.

#### iPSC-CM culture and differentiation

We used the episomal hiPSC line A18944 (Thermo Fisher Scientific). hiPSCs were stored in liquid nitrogen at -196°C prior to use and revived by rapidly thawing at 37°C before gently adding 10 mL growth medium ((mTeSR™1 basal medium (STEMCELL Technologies) supplemented with 20% mTeSR™1 5X supplement (STEMCELL Technologies)). Cells were then centrifuged at 200 x *g* for 2 min, the supernatant was removed and cells were resuspended in 1 mL growth medium. Each well of ice-cold 6-well CytoOne plates (STARLAB) was coated with 1.5 mL Dulbecco’s modified Eagle medium (DMEM) (Thermo Fisher Scientific) containing 1% Matrigel® growth factor reduced basement membrane matrix (Corning) and incubated at 37°C/5% CO_2_ for 1 h. The matrigel mix was then removed from the vessel and replaced with 2 mL growth medium. Cells were then seeded onto the coated 6-well plates and maintained at 37°C in the presence of 5% CO_2_ in air and growth medium was replaced every day. Cells were grown to 80% confluency and passaged at a ratio of 1:12 by washing with 1 mL Dulbecco’s phosphate buffered saline (PBS) (Sigma-Aldrich), detaching with 1 mL ReLeSR™ (STEMCELL Technologies) for 3 min at 37°C and resuspending in growth medium containing 2 μM thiazovivin (Sigma-Aldrich) to avoid apoptosis. iPSCs were passaged at a ratio of 1:12 and cultured for 4 d prior to commencing differentiation. On differentiation day 0, growth medium was removed and replaced with 2 mL CDM3 medium ((RPMI 1640 basal medium, high glucose (Thermo Fisher Scientific) supplemented with 64 mg/mL L-ascorbic acid 2-phosphate (Sigma-Aldrich), 75 mg/mL recombinant human albumin (Sigma-Aldrich) and 50 U/mL penicillin-streptomycin (Thermo Fisher Scientific)) plus 6 μM CHIR99021 (Sigma-Aldrich) for 48 h. On day 2, the medium was removed and replaced with CDM3 medium containing 2 μM WntC59 (Cayman Chemical) for 48 h. On day 4, the medium was removed and replaced with B27 medium ((RPMI 1640 basal medium, high glucose supplemented with 2% B-27™ Supplement, minus insulin (Thermo Fisher Scientific) and 50 U/mL penicillin-streptomycin) for 48 h. B27 medium was replaced every 48 h thereafter until cells were visibly beating around day 10.

#### H9C2 culture

H9c2 rat cardiomyoblasts (ATCC) were stored at -196°C in liquid nitrogen prior to rapid thawing in a 37°C waterbath for 2 min. 1 mL thawed cell solution was diluted with 9 mL culture media (90% DMEM, 10% FBS, 1% 100X MEM non-essential amino acids solution (Gibco) and 100 units/mL penicillin and streptomycin) and centrifuged at 1000 rpm for 5 min. The media was discarded, and the cell pellet was resuspended in fresh culture media and seeded onto 75 cm^2^ T75 flasks (Corning) and maintained in a 37°C/5% CO_2_ incubator. Culture media was renewed every 2-3 days. Upon reaching ≤80% confluency cells were passaged at a ratio of 1:4 by removing the culture media, detaching the cells with 3 mL trypsin-EDTA solution for 2 min, inactivating the trypsin by addition of 9 mL fresh culture media and distributing 3 mL of detached cell solution into T75 flasks containing pre-warmed culture media. For experimental work, H9c2 cells were seeded onto 6-well plates at a density of 3 x 10^5^ cells/well or onto 24-well plates at a density of 5 x 10^4^ cells/well.

#### NIH/3T3 culture

NIH/3T3 fibroblasts (ATCC) were stored at -196°C in liquid nitrogen prior to rapid thawing in a 37°C waterbath for 2 min. 1 mL thawed cell solution was diluted with 9 mL culture media (90% DMEM, 10% FBS and 100 units/mL penicillin and streptomycin) and centrifuged at 1000 rpm for 5 min. The media was discarded, and the cell pellet was resuspended in fresh culture media and seeded onto 75 cm^2^ T75 flasks (Corning) and maintained in a 37°C/5% CO_2_ incubator. Culture media was renewed every 2-3 days. Upon reaching ≤80% confluency cells were detached by trypsin-EDTA as described above and cultured in DMEM supplemented with 10% FBS and 100 units/mL penicillin and streptomycin. For experimental work, NIH/3T3 cells were seeded onto 6-well plates at a density of 3 x 10^5^ cells/well or onto 24-well plates at a density of 5 x 10^4^ cells/well.

#### Adenovirus mediated gene overexpression

Adenovirus (Ad) vectors used herein to induce overexpression of target proteins were generated using the pENTR™:pAd/CMV/V5-DEST™ Gateway® vector system (Thermo Fisher Scientific). Cells were seeded onto 6-well or 24-well plates and infected with Ad-FLAG-5-HT_2B_ or Ad-LacZ for 48 h to induce overexpression of the 5-HT_2B_ receptor or control β-Galactosidase, respectively. For luciferase assay experiments, cells were subsequently infected for a further 24 h with Ad-GAL4-TEAD and Ad-UAS-luciferase. The Ad-GAL4-TEAD was generated from GAL4-TEAD plasmid, which was a gift from Kunliang Guan (Addgene plasmid # 24640; http://n2t.net/addgene:24640; RRID:Addgene_24640). The Ad-UAS-luciferase was generated from pUAS-luc2, which was was a gift from Liqun Luo (Addgene plasmid # 24343; http://n2t.net/addgene:24343; RRID:Addgene_24343). For YAP localisation experiments, cells were infected with Ad-GFP-YAP, generated from the plasmid construct pEGFP-C3-hYAP1, which was a gift from Marius Sudol (Addgene plasmid # 17843; http://n2t.net/addgene:17843; RRID:Addgene_17843).

#### YAP luciferase assay

NRCMs were seeded onto 24-well plates at a density of 2.5 x 10^5^ cells/well and infected with either Ad-FLAG-5-HT_2B_ or Ad-LacZ for 48 h. The media was then replaced and NRCMs were infected for a further 24 h with Ad-GAL4-TEAD and Ad-UAS-luciferase. Cells were washed once with PBS and lysed with 100 μL 1X cell lysis buffer (Promega) on an orbital shaker for 20 min at 4°C. 20 μL cell lysate was combined with 80 μL luciferase assay reagent (Promega) and the luminescence, proportional to the transcriptional activity of YAP, was measured immediately using a tube luminometer (Lumat LB9507). In each independent experiment, mean luminescence was calculated from five wells per treatment group.

#### Hydrogen peroxide-induced oxidative stress

NRCMs were infected with adenoviruses as described above and after 48 h the infection media was replaced with maintenance media supplemented with 100 μM hydrogen peroxide (H_2_O_2_) or an equivalent volume of sterile water as a control and the cells were incubated at 37°C/5% CO_2_ for 2 h to in order to provide an *in-vitro* model of oxidative stress.

#### Immunofluorescence experiments

For *in-vitro* immunofluorescence experiments, NRCMs were seeded onto 24-well plates containing UV-sterilised coverslips precoated with 5 μg/cm^2^ laminin (Roche #11243217001) in PBS. Following gene infection described above (and treatment with H_2_O_2_ for TUNEL assay experiments), cells were washed three times in PBS and fixed in 4% paraformaldehyde (PFA) (Fisher) in PBS for 20 min at RT. Cells were then permeabilised in 0.5% Triton X-100 (Sigma-Aldrich) for 20 min, blocked for 1 h in 1% bovine serum albumin (BSA) (Sigma-Aldrich) in PBS and probed with primary antibodies (1:100) in 1% BSA in PBS overnight at 4°C. Following overnight incubation, cells were washed three times in PBS and incubated with secondary antibodies (1:200) in 1% BSA in PBS for 2 h at RT in the dark. Cells were then washed three times in PBS and incubated with 4ʹ,6-diamidino-2-phenylindole (DAPI) (Invitrogen) in PBS (1:5000) for 1 min at RT. Cells were washed a further three times in PBS and once in distilled water then mounted to Permafrost™ microscope slides (Cardinal Health) using VECTASHIELD® antifade mounting media (Vector Laboratories). Coverslips were visualised on an upright fluorescence microscope (Zeiss Axioimager) and 5 to 10 random images per coverslip were captured under a 10X or 20X objective and the images were then analysed on ImageJ software (NIH).

#### YAP nuclear translocation assay

NRCMs were seeded onto laminin-coated coverslips in 24-well plates at a density of 2.5 x 10^5^ cells/well and infected with either Ad-FLAG-5-HT_2B_ or Ad-LacZ for 48 h after which the media was replaced and cells were infected for a further 24 h with Ad-GFP-YAP. The cells were then washed, fixed, counterstained with DAPI, mounted and imaged as described above. GFP positive nuclei indicating YAP nuclear localisation and by extension YAP activity were counted using ImageJ and the percentage of GFP+ nuclei to total number of nuclei was calculated for each image. In each independent experiment, 5 random images were captured per coverslip under a 10X objective and 3 coverslips per group were imaged. The mean percentage of GFP+ nuclei was calculated for each coverslip and the mean of 3 coverslips per group was calculated per independent experiment.

#### Detection of Ki67

NRCMs were seeded onto laminin-coated coverslips in 24-well plates at a density of 2.5 x 10^5^ cells/well and infected with either Ad-FLAG-5-HT_2B_ or Ad-LacZ for 48 h. The cells were then washed, fixed, permeabilised and blocked as described and probed with mouse anti-α-actinin and rabbit anti-Ki67 primary antibodies overnight. The following day cells were washed and incubated with anti-mouse AlexaFluor® 647 and anti-rabbit Alexa Fluor® 488 secondary antibodies and then washed, counterstained with DAPI, mounted and imaged as described. Ki67 is detectable during all active phases of the cell cycle but is absent in non-proliferating cells, while α-actinin is present in cardiomyocytes but absent in non-muscle cardiac cells. Cardiomyocytes with Ki67 positive nuclei were counted using ImageJ and the percentage of Ki67+ cardiomyocytes to total number of cardiomyocytes was calculated for each image. In each independent experiment 5 random images were captured per coverslip under a 10X objective and 3 coverslips per group were imaged. The mean percentage of Ki67+ cardiomyocytes was calculated for each coverslip and the mean of 3 coverslips per group was calculated per independent experiment.

#### EdU incorporation assay

NRCMs were seeded onto laminin-coated coverslips in 24-well plates at a density of 2.5 x 10^5^ cells/well and infected with either Ad-FLAG-5-HT_2B_ or Ad-LacZ for 48 h. Cells were then incubated with 5 μM 5-ethynyl-2′-deoxyuridine (EdU) (Thermo Fisher Scientific), a nucleoside analogue of thymidine that is incorporated into DNA during active DNA synthesis of cells in interphase. Following EdU incorporation, cells were washed, fixed and permeabilised as described and the incorporated EdU was labelled according to the Click-iT EdU Cell Proliferation Kit for Imaging (Thermo Fisher Scientific) manufacturer instructions. Briefly, cells were washed twice with 3% BSA in PBS then incubated with Click-iT® reaction cocktail for 30 min at RT in the dark. The reaction cocktail contained Alexa Fluor® 488 dyes containing azide groups, which form covalent bonds with EdU alkyne groups in the presence of a copper catalyst, thereby fluorescently labelling replicating DNA in the nuclei of proliferating cells. The cells were then washed again in 1% BSA in PBS, blocked for 1 h and probed with mouse anti-α-actinin primary antibody overnight, then washed and incubated with anti-mouse AlexaFluor® 647 secondary antibody, washed, counterstained with DAPI, mounted and imaged as described. Cardiomyocytes with EdU positive nuclei were counted using ImageJ and the percentage of EdU+ cardiomyocytes to total number of cardiomyocytes was calculated for each image. In each independent experiment 5 random images were captured per coverslip under a 10X objective and 3 coverslips per group were imaged. The mean percentage of EdU+ cardiomyocytes was calculated for each coverslip and the mean of 3 coverslips per group was calculated per independent experiment.

#### Detection of phospho-histone H3

NRCMs were seeded onto laminin-coated coverslips in 24-well plates at a density of 2.5 x 10^5^ cells/well and infected with either Ad-FLAG-5-HT_2B_ or Ad-LacZ for 48 h. The cells were then washed, fixed, permeabilised and blocked as described and probed with mouse anti-α-actinin and rabbit anti-phosphohistone H3 (PHH3) primary antibodies overnight at 4°C. The following day cells were washed and incubated with anti-mouse AlexaFluor® 647 and anti-rabbit Alexa Fluor® 488 secondary antibodies and then washed, counterstained with DAPI, mounted and imaged as described. PHH3 is detectable specifically during mitosis and is negligible in the other phases of the cell cycle. Cardiomyocytes with PHH3 positive nuclei were counted using ImageJ and the percentage of PHH3+ cardiomyocytes to total number of cardiomyocytes was calculated for each image. In each independent experiment 5 random images were captured per coverslip under a 10X objective and 3 coverslips per group were imaged. The mean percentage of PHH3+ cardiomyocytes was calculated for each coverslip and the mean of 3 coverslips per group was calculated per independent experiment.

#### TUNEL assay

NRCMs were seeded onto laminin-coated coverslips in 24-well plates at a density of 2.5 x 10^5^ cells/well and infected with either Ad-FLAG-5-HT_2B_ or Ad-LacZ for 48 h. The media was then replaced with maintenance media supplemented with 100 μM hydrogen peroxide (H_2_O_2_) or water control and the cells were incubated at 37°C/5% CO_2_ for 2 h. The cells were then washed and fixed as described and permeabilised in 0.1% Triton X-100 and 0.1% sodium citrate for 8 min then washed twice with PBS. Terminal deoxynucleotidyl transferase (TdT) deoxyuridine triphosphate (dUTP) Nick-End Labeling (TUNEL) was used to label DNA breaks in apoptotic cells according to the *In Situ* Cell Death Detection Kit instructions. Briefly, cells were treated with fluorescein-conjugated dUTP, which labels the 3’-OH ends of DNA breaks in a reaction catalysed by TdT, for 1 h at 37°C. The cells were then washed in 1% BSA in PBS, blocked for 1 h and probed with mouse anti-α-actinin primary antibody overnight, then washed and incubated with anti-mouse AlexaFluor® 647 secondary antibody, washed, counterstained with DAPI, mounted and imaged as described. Cardiomyocytes with TUNEL positive nuclei were counted using ImageJ and the percentage of TUNEL+ cardiomyocytes to total number of cardiomyocytes was calculated for each image. In each independent experiment 5 random images were captured per coverslip under a 10X objective and 3 coverslips per group were imaged. The mean percentage of TUNEL+ cardiomyocytes was calculated for each coverslip and the mean of 3 coverslips per group was calculated per independent experiment.

#### Genotyping of transgenic mice

Ear tissue biopsies were taken from mouse litters and incubated in 200 μL lysis buffer (50 mM Tris pH 8, 100 mM EDTA, 0.5% sodium dodecyl sulfate (SDS) (Sigma-Aldrich)) supplemented with 10 μL proteinase K (Sigma-Aldrich) overnight at 56°C. Samples were centrifuged at 13,000 rpm for 10 min at RT, the supernatant was transferred to a fresh tube and DNA was precipitated by incubation with 200 μL propan-2-ol (Thermo Fisher Scientific) for 1 h on ice. Samples were then centrifuged at 13,000 rpm for 10 min at 4°C and the supernatant was discarded. The DNA pellet was washed with 500 μL 70% ethanol in water and centrifuged again at 13000 rpm for 5 min at 4°C. The ethanol was then removed, and the pellets were air dried and resuspended in 50 μL nuclease-free water. DNA concentration was measured using a NanoDrop2000 (Thermo Fisher Scientific) and stored at 4°C.

Polymerase chain reaction (PCR) was performed to amplify a sequence of 474 base pairs present in the genome of 5-HT_2B_^cTG^ mice but absent in WT littermates. For each DNA sample, 12.5 μL Phusion Green Hot Start II High-Fidelity PCR Master Mix (Thermo Fisher Scientific) was mixed with 1 μL forward primer (5’-TGTAATCTTGATGAATGCAGTAGCC-3’), 1 μL reverse primer (5’ CAGAAGACATGTGATCACCTGATC-3’) (Merck), 100 ng DNA and nuclease-free water to a final volume of 25 μL. PCR was then performed on a Veriti™ 96-well thermal cycler (Applied Biosystems). PCR products were then run alongside HyperLadder™ 1kb (Bioline) on a 1% agarose gel in TAE buffer (40 mM Tris base, 20 mM acetic acid, 1mM EDTA) containing 0.06 μL/mL midori green (Geneflow Limited) at 100 V for 40 min and visualised using the ChemiDoc™ XRS+ imaging system (BioRad).

#### Intracardiac modRNA injection model

The *in-vivo* role of 5-HT_2B_ in the heart following MI was also investigated using 12-week-old male and female WT C57BL/6J mice (Envigo) injected with modified RNA constructs containing either the mouse Htr2b mRNA sequence or luciferase mRNA control. Mice were supplied to the University of Manchester Biological Services Facility at 8 weeks old and housed under a 12 h light/dark cycle at 19-22°C and 40-65% humidity with a standard chow diet. To synthesise the modRNA constructs, DNA plasmid templates were first generated through restriction cloning, validated by sanger sequencing and linearised prior to *in-vitro* transcription (IVT) using commercial RNA synthesis kits with certain key nucleoside modifications detailed below. modRNA transcripts were then polyadenylated, purified and validated *in-vitro* and *in-vivo* prior to commencing MI experiments.

#### Generation of modRNA

DNA templates were generated via restriction cloning using the pENTR11™:pcDNA6.2™ Gateway® vector system (Thermo Fisher Scientific). 4 μg pHtr2b (Origene #MR224713), pGL3 luciferase reporter (Promega #E1751) and pENTR11™ (Thermo Fisher Scientific) were each digested with 20 units (U) of the restriction enzymes (RE) KpnI-XhoI (pGL3 + pENTR11) or KpnI-NotI (pHtr2b + pENTR11) in the presence of 1X CutSmart buffer (NEB) in a final volume of 40 μL nuclease-free water per reaction overnight at 37°C. The digested DNA products were run on a 1% agarose gel in TAE buffer containing 0.06 μL/mL midori green at 100 V for 30 min. The gels were visualised under a UV light and the desired DNA bands were cut out of the gel and purified using a QIAquick gel extraction kit (Qiagen) following manufacturer instructions. Briefly, the excised gel was weighed and dissolved in kit buffer at 50°C for 10 min. DNA was precipitated with isopropanol and bound to a spin column by centrifugation, washed and eluted in 20 μL nuclease-free water. Htr2b and GL3 luciferase were each ligated into pENTR11™ at a ratio of 1:1 in a reaction catalysed by 1 U T4 DNA ligase (Promega) in 1 μL T4 ligase buffer (Promega) and nuclease-free water to a volume of 10 μL per reaction overnight at 25°C. pENTR11:Htr2b and pENTR11:luciferase ligation reactions were added to 35 μL DH5α competent *E.coli* (Thermo Fisher Scientific) and incubated on ice for 35 min. The cells were then subjected to heat shock in a 42°C waterbath for 150 s then added to 100 μL sterile LB broth (Sigma-Aldrich) and incubated for 1 h in a shaking incubator (VWR) at 150 rpm at 37°C. The bacteria were then transferred to LB-agar (Sigma-Alrich) plates containing 50 μg/mL kanamycin (Sigma-Alrich) and incubated overnight at 37°C. Colonies were selected and cultured in 250 mL LB broth supplemented with 50 μg/mL kanamycin overnight at 150 rpm/37°C. DNA was then isolated from overnight bacterial cultures using a PureLink™ HiPure Plasmid Maxiprep Kit (Invitrogen) following manufacturer instructions. Briefly, cell culture was pelleted by centrifugation at 4000 x *g* for 10 min at RT then resuspended and lysed with kit buffer for 5 min. Precipitation buffer was then added and the mixture was centrifuged at 12,000 x *g* for 10 min at RT and the supernatant containing the DNA fraction was loaded and bound onto a column by gravity flow, washed and eluted in 500 μL nuclease-free water and stored at -20°C. pENTR11:Htr2b and pENTR11:luciferase entry clones were inserted into pcDNA6.2/V5-DEST destination vector by combining 150 ng entry clone and 150 ng destination vector with 2 μL LR Clonase® II (Thermo Fisher Scientific) in nuclease-free water to a volume of 10 μL per reaction and incubating the reactions at 25°C overnight. 1 μL proteinase K was added to terminate the reaction and the pcDNA6.2/V5:Htr2b and pcDNA6.2/V5:luciferase plasmids were amplified by heat shock mediated transformation and clonal expansion with DH5α competent *E.coli* as described above. Sequence fidelity of the DNA products was verified by sanger sequencing at the University of Manchester Genomic Technologies Core Facility.

10 μg pcDNA6.2/V5:Htr2b and pcDNA6.2/V5:luciferase DNA plasmids were linearised with 50 U Xho1 in the presence of 1X CutSmart buffer in a final volume of 30 μL nuclease-free water per reaction overnight at 37°C. 60 μL isopropanol was added to terminate the reaction and samples were incubated on ice for 15 min, then centrifuged at 13,000 rpm for 1 min at 4°C. The supernatant was carefully removed and the linearised plasmids were resuspended in nuclease-free water to a concentration of 1 μg/μL.

*In vitro* transcription (IVT) reaction was carried out on linear template DNA using the MEGAscript® T7 Kit (Invitrogen) with important nucleoside modifications to enhance RNA stability and translation efficiency. The composition of IVT reaction components is detailed in Table S1. 20μL IVT reactions were incubated for 4 h at 37°C.

The *in-vitro* transcribed RNA was polyadenylated to further enhance stability and translation efficiency using the Poly(A) Tailing Kit (Invitrogen). The components of polyA tailing reaction are detailed in Table S2 for 1 h at 37°C.

modRNA was purified using a MEGAclear™ Transcription Clean-Up Kit following manufacturer instructions. Briefly, 350 μL kit binding solution and 250 μL ethanol was added to each modRNA sample and the mixture was applied to a filter spin column and centrifuged at 12,000 x *g* for 1 min at RT. The bound RNA was washed twice with wash solution and 50 μL elution solution was then applied to the filter, incubated on a heat block at 70°C for 5 min and centrifuged at 12,000 x *g* for 1 min at RT to collect the modRNA. This was repeated with a further 50 μL elution solution to maximise RNA recovery to give 100 μL modRNA product per reaction.

12 μL 10X Antarctic phosphatase enzyme (NEB) and 12 μL 10X Antarctic phosphatase buffer (NEB) was added to each modRNA sample and incubated for 1 h at 37°C to dephosphorylate the 5-' and 3-' ends of the RNA and remove unincorporated nucleotides. 12 μL 5M ammonium acetate (Invitrogen) and 330 μL ethanol was then added to the samples and incubated at -20°C overnight. The following day samples were centrifuged at 14,000 rpm for 15 min at 4°C, the supernatant was discarded, and the RNA was washed with 500 μL 70% ethanol in nuclease-free water and centrifuged again at 14,000 rpm for 15 min at 4°C. The RNA pellet was airdried and resuspended in 30 μL nuclease-free water and the RNA concentration was measured using a NanoDrop2000. For *in-vivo* injections, modRNA samples were pooled, repurified and eluted in nuclease-free water to a concentration of 5 μg/μL.

#### *In-vitro* validation of modRNA mediated expression

modRNA-Htr2b and modRNA-Luciferase constructs were first validated *in-vitro* using a luciferase assay and western blot. H9c2 cells were seeded onto 6-well plates at a density of 3 x 10^5^ cells/well or onto 24-well plates at a density of 5 x 10^4^ cells/well and grown to 50% confluency. For each well of a 6-well plate, 30 pmol modRNA was diluted in 250 μL OptiMEM, while in a separate tube 7.5 μL Lipofectamine® RNAiMAX (RNAiMAX) (Invitrogen) was diluted in 250 μL OptiMEM and incubated at RT for 5 min. For each well of a 24-well plate, 5 pmol modRNA was diluted in 50 μL OptiMEM, while in a separate tube 1.5 μL RNAiMAX was diluted in 50 μL OptiMEM and incubated at RT for 5 min. The diluted modRNA and RNAiMAX were then combined and incubated at RT for 5 min. The modRNA/RNAiMAX complexes were then added in a dropwise manner to the cells and incubated at 37°C/5% CO_2_ for 24 h (24-well plates) or 48 hours (6-well plates).

For Luciferase Assay**,** H9c2 cells were seeded onto 24-well plates at a density of 5 x 10^4^ cells/well, grown to 50% confluency and transfected with 5 pmol modRNA-Htr2b or modRNA-Luciferase for 24 h. Cells were washed once with PBS and lysed with 100 μL 1X cell lysis buffer on an orbital shaker for 20 min at 4°C. 20 μL cell lysate was combined with 80 μL luciferase assay reagent and the luminescence was measured immediately using a tube luminometer for five wells per treatment group.

For Western blot, H9c2 cells were seeded onto 6-well plates at a density of 3 x 10^5^ cells/well, grown to 50% confluency and transfected with 30 pmol modRNA-Htr2b or modRNA-Luciferase for 48 h. Cells were washed once with PBS and lysed with 100 μL RIPA buffer (PBS containing 1% IGEPAL CA-630, 0.5% sodium deoxycholate, 0.1% SDS, 0.5 mM phenylmethylsulfonyl fluoride (PMSF), 1 μg/mL aprotinin, 2.5 μg/mL pepstatin A, 500 ng/mL leupeptin) (Sigma-Aldrich) for 20 min at 4°C. The lysate was then centrifuged at 8000 rpm for 6 min at 4°C and the supernatant was transferred to a clean tube and stored at -20°C. Protein expression of 5-HT_2B_ was determined by western blot and normalised to the housekeeper GAPDH. Expression was determined in three wells per treatment group.

#### *In-vivo* validation

modRNA-Htr2b and modRNA-Luciferase constructs were validated *in-vivo* in 12-week-old female C57BL/6J mice prior to beginning MI experiments. 50 μg modRNA constructs were diluted in 5 μL OptiMEM while in separate tube 10 μL RNAiMAX was diluted in 5 μL OptiMEM and incubated at RT for 5 min. The diluted modRNA was then added to the RNAiMAX and incubated for a further 20 min at RT. The modRNA/RNAiMAX complexes were then drawn up through a 31G insulin needle (BD) into a sterile syringe to a total volume of 30 μL per syringe. General anaesthesia was induced in mice by inhalation of 5% isoflurane in oxygen (Baxter). Analgesia was delivered by subcutaneous injection of 0.1 mg/kg buprenorphine (Vetergesic) and anaesthesia was maintained at 3% isoflurane in oxygen throughout surgery. Following minithoracotomy to expose the heart, the entire 30 μL modRNA solution containing 50 μg modRNA was injected into the LV myocardium. The chest was then sutured closed, and the mice were administered 200 μL saline by intraperitoneal injection and allowed to recover in a 32°C incubator and returned to normal housing after 6 h provided with mashed food and water. After 48 h, general anaesthesia was induced and maintained with 3% isoflurane in oxygen and the mice were administered 200 μL intraperitoneal injection of 15 mg/mL VivoGlo™ luciferin (Promega) and the bioluminescence was measured using the IVIS® Spectrum *in vivo* imaging system (Perkin Elmer) for 40 min. The mice were then terminated via cervical dislocation and the hearts were removed, washed in PBS and snap frozen in liquid nitrogen. Each frozen heart was homogenised in 500 μL RIPA buffer using a Dounce homogeniser and incubated at 4°C for 20 min. The lysates were then centrifuged at 8000 rpm for 6 min at 4°C and the supernatant was transferred to a clean tube and stored at -20°C. Protein expression of 5-HT_2B_ was determined by western blot and normalised to the housekeeper GAPDH.

#### 5-HT_2B_^cTG^ MI experiments

To investigate the role of chronic overexpression of 5-HT_2B_ in response to MI, LAD ligation was conducted on 12-week-old male and female 5-HT_2B_^cTG^ TG mice and their WT littermates, alongside sham operated controls in which the chest was opened but the LAD was not ligated, and the animals were retained 4 weeks.

#### modRNA MI experiments

To investigate the role of transient acute overexpression of 5-HT_2B_ in response to MI, LAD ligation was conducted on 12-week-old male and female C57BL/6J WT mice followed immediately by intracardiac injection of 50 μg modRNA-Htr2b or modRNA-Luciferase, and the animals were retained for 4 weeks.

#### Ipsapirone IR experiments

To investigate the role of the serotenergic compound ipsapirone in response to ischaemia-reperfusion injury, the LAD was ligated as for MI experiments however the ligation was removed after 45 min in 12-week-old female C57BL/6J WT mice followed by daily intraperitoneal injections of 5 mg/kg ipsapirone and the animals were retained for 2 weeks.

#### LAD ligation

General anaesthesia was induced in 12-week-old mice by inhalation of 5% isoflurane in oxygen. Analgesia was then delivered by subcutaneous injection of 0.1 mg/kg buprenorphine and the mice were intubated and placed on a ventilator at 200 breaths per minute and a tidal volume of 0.1 mL (Minivent 845, Harvard Apparatus) and anaesthesia was maintained at 3% isoflurane in oxygen throughout surgery. To summarise, a 5 mm incision was made in the skin at the left sternal border and left minithoracotomy was performed through the 4th intercostal space to expose the heart, and the LAD coronary artery was permanently ligated with an 8-0 nylon suture (ETHILON). MI was visually confirmed by a colour change in the LV myocardium from pinkish red to pale. For modRNA experiments, 30 μL modRNA solution containing 50 μg modRNA-Htr2b or modRNA-Luciferase was injected into the LV myocardium at this point. In sham-operated controls the LAD was not ligated. The chest was then sutured closed with a 6-0 prolene suture (ETHILON) and mice were administered 200 μL saline by intraperitoneal injection, allowed to recover in a 32°C incubator, then returned to normal housing and provided with mashed food and water for up to 3 days.

#### Determination of plasma cardiac troponin I

24 h following LAD ligation, 5% EMLA cream (AstraZeneca) containing lidocaine and prilocaine was applied to the tails of the mice and allowed to absorb for 20 min to provide local anaesthesia. The mice were then restrained in a rodent restraint tube placed on a heat mat and 7.5% povidone-iodine cutaneous solution (Videne) was applied to the tail and to a scalpel prior to making a small incision in the lateral tail vein. 40 μL blood was collected from the tail vein and mixed immediately with ice-cold 3.2% sodium citrate (Sigma-Aldrich) to prevent coagulation. Blood samples were centrifuged at 8000 rpm for 6 min at 4°C and the plasma supernatant was transferred to a clean tube and stored at -80°C.

Plasma titres of cardiac troponin I (cTnI) were determined using a commercial mouse cardiac troponin-I enzyme linked immunosorbent assay (ELISA) CTNI-1-HSP kit (Life Diagnostics) following manufacturer instructions. Briefly, an 8-point standard curve was prepared by serial dilution of kit standard and plasma samples were diluted 1:4 with kit diluent. Standards and samples were loaded onto a 96-well ELISA plate pre-coated with anti-mouse cTnI antibody and horseradish peroxidase (HRP)-linked secondary antibody was then added to the wells and incubated on an orbital shaker at 150 rpm for 1 h at RT. The wells were then washed and the HRP substrate tetramethylbenzidine (TMB) was added to the wells and incubated at 150 rpm for 20 min at RT. Finally, stop solution was added to the wells and the absorbance at 450 nm was measured using a Multiskan ascent spectrophotometer. The absorbance values of the standards were plotted against their known log_10_ concentrations, and a standard curve was fitted with a 4-parameter logistic regression equation. Sample cTnI concentrations were derived from the antilog of their x values interpolated from the standard curve and multiplied by 4.

#### Echocardiography

To assess the structural dimensions of the mouse heart and to quantify cardiac systolic and diastolic performance, transthoracic two-dimensional echocardiography was performed using a Vevo770 ultrasound machine fitted with a 14 MHz transducer (Visualsonics). Echocardiography was carried out at week 1 and week 4 post-MI. General anaesthesia was induced by 2% isoflurane in oxygen and maintained at 1% isoflurane in oxygen. Fur was removed from the chest area by hair removal cream allowing ultrasound emission gel to be applied to the bare chest. M-mode ultrasound images of the heart were generated in the parasternal short-axis view and measurements were recorded for i) LV diameter, ii) interventricular septal (IVS) thickness, and iii) posterior wall (PW) thickness in both diastole (*d*) systole (*s*). From these measurements, ejection fraction (EF) and LV mass were calculated using the formulas outlined in Table S3.

#### Tissue collection

Mice were terminated by cervical dislocation 4 weeks post-MI. The hearts were quickly extracted, washed in PBS, weighed, and placed in 4% PFA in PBS overnight at 4°C if used for histological analysis, or washed in PBS and snap frozen in liquid nitrogen and stored at -80°C if used for molecular analysis. In addition, the body weights and heart weights of the mice were recorded.

#### Histological analyses

Mouse hearts were collected 4 weeks following MI and fixed in 4% PFA in PBS overnight at 4°C. The following day, hearts were transferred to 70% ethanol in water and stored at 4°C for at least 24 h. Tissue was then processed overnight using an automated tissue processor (Leica). Following processing, each heart was embedded in paraffin wax. 5 μm tissue sections were then prepared from six levels of the heart using an automated microtome (Leica 2255), with the first level defined as the apex of the heart and with a distance of 500 μm between each subsequent level. Sections were mounted on poly-L-lysine coated slides (VWR), dried for 24 h in a 37°C incubator and stored at RT.

#### Masson’s trichrome staining

Tissue sections from 6 levels of the heart were deparaffinised on a heat block at 80°C for 2 min and cleared in xylene (Thermo Fisher Scientific) for 1 h. Sections were then sequentially rehydrated in decreasing percentages of industrial methylated spirit (IMS) (Thermo Fisher Scientific) in water (100%, 90%, 75%) and washed in distilled water. Sections were immersed in Bouin’s solution (Sigma-Aldrich) for 2 h, washed in distilled water and placed in filtered Haematoxylin solution (Sigma-Aldrich) for 5 min to stain the nuclei black. The sections were then washed in distilled water and differentiated in 1% hydrochloric acid in ethanol for 10 s prior to immersion in Biebrich Scarlet-Acid Fuchsin solution (GCC Diagnostics) for 5 min to stain the myocardium red. The slides were then washed again and treated with 2.5% phosphomolybdic acid for 30 minutes to chelate nonspecific red staining and then immersed in Aniline blue solution for 5 minutes to stain collagen fibres blue. Sections were rinsed briefly in distilled water and differentiated in 1% acetic acid solution for 2 min. Finally, the sections were sequentially dehydrated in increasing percentages of IMS in water (75%, 90%, 100%), cleared in xylene and mounted under a coverslip using dibutylphthalate polystyrene xylene (DPX) mounting medium (Sigma-Aldrich). Once dried overnight, sections were imaged on a 3D Histech Panoramic 250 Flash II slide scanner.

#### Determination of infarct size

Infarct sizes of MI hearts were determined with a length-based measurement approach using CaseViewer (3DHISTECH) to analyse Masson’s trichrome stained sections. Epicardial and endocardial circumference, and epicardial and endocardial infarct length were measured on sections over 6 levels of the heart and infarct size was determined by averaging the epicardial infarct ratio (the sum of 6 epicardial infarct lengths divided by the sum of 6 epicardial circumferences) and the endocardial infarct ratio (the sum of 6 endocardial infarct lengths divided by the sum of 6 endocardial circumferences) and multiplying by 100.

Infarct areas of I/R hearts, which retain significantly more muscularisation compared to permanent LAD ligation, were calculated by averaging the fibrotic area of each section as a percentage of the whole tissue area using the ImageJ colour threshold function to quantify red areas of fibrosis of picrosirius red stained sections.

#### Determination of cell area

CaseViewer was used to determine the cardiomyocyte area in μm^2^ of 100 randomly selected cells from the LV using heart sections prepared from the same location of the heart. The mean cardiomyocyte area was then calculated for each mouse heart.

#### TUNEL staining of histological sections

Tissue sections from level 3 of the heart were deparaffinised on a heat block at 80°C for 2 min and cleared in xylene for 1 h. Sections were then sequentially rehydrated in decreasing percentages of industrial IMS in water (100%, 90%, 75%) and washed in distilled water. Sections were immersed in 3% H_2_O_2_ for 15 min and washed in distilled water for 20 min followed by PBS for 10 min. The sections were then permeabilised with 20 μg/mL proteinase K in PBS for 15 min at 37°C followed by 0.1% Triton X-100 and 0.1% sodium citrate for 8 min at RT. Sections were washed twice with PBS and TUNEL staining was performed to label DNA breaks in apoptotic nuclei using an in-situ cell death detection kit. Briefly, fluorescein-conjugated dUTP labelled the 3’-OH ends of DNA breaks in a reaction catalysed by TdT for 1 h at 37°C. Sections were then blocked with 1% BSA in PBS for 1 h at RT and probed with mouse anti-α-actinin primary antibody (1:100) overnight at 4°C. The following day the sections were washed and incubated with anti-mouse AlexaFluor® 647 secondary antibody (1:200) for 2 h at RT in the dark, then washed, counterstained with DAPI (1:5000), washed again and mounted under a coverslip using ProLong™ Gold Antifade Mountant (Thermo Fisher Scientific). Once dried overnight, sections were imaged on a 3D Histech Panoramic 250 Flash II slide scanner.

#### Determination of apoptosis

CaseViewer was used to capture up to 10 random images of sections at level 3 of the heart at 20X magnification and the acquired images were then analysed using ImageJ. TUNEL positive nuclei were counted and the percentage of TUNEL+ nuclei to total number of nuclei was calculated for each image. The mean percentage of TUNEL+ nuclei was then calculated for each animal.

#### Ki67 staining of histological sections

Tissue sections from level 3 of the heart were deparaffinised on a heat block at 80°C for 2 min and cleared in xylene for 1 h. Sections were then sequentially rehydrated in decreasing percentages of industrial IMS in water (100%, 90%, 75%) and washed in distilled water. Sections were immersed in sodium citrate buffer (10 nM sodium citrate, 0.05% Tween 20; pH 6.0) (Sigma-Aldrich), secured in a pressure cooker and microwaved for 20 min. The sections were brought to RT, washed in distilled water and permeabilised in 0.1% Triton X-100 for 30 min, then blocked with 1% BSA in PBS for 1 h at RT. Tissue sections were then probed with mouse anti-α-actinin (1:100) and rabbit anti-Ki67 primary antibodies (1:100) overnight at 4°C. The following day the sections were washed and incubated with anti-mouse AlexaFluor® 647 and anti-rabbit AlexaFluor® 488 secondary antibodies (1:200) for 2 h at RT in the dark, then washed, counterstained with DAPI (1:5000), washed again and mounted under a coverslip using ProLong™ Gold Antifade Mountant. Once dried overnight, sections were imaged on a 3D Histech Panoramic 250 Flash II slide scanner.

#### Determination of proliferation

CaseViewer was used to capture up to 10 random images of sections at level 3 of the heart at 20X magnification and the acquired images were then analysed using ImageJ. Ki67 positive nuclei were counted and the percentage of Ki67+ nuclei to total number of nuclei was calculated for each image. The mean percentage of Ki67+ nuclei was then calculated for each animal.

#### Active YAP immunohistochemistry

5 μm tissue sections prepared from modRNA-injected mouse hearts were deparaffinised on a heat block at 80°C for 2 min and cleared in histoclear for 10 min. Sections were then sequentially rehydrated in decreasing percentages of industrial methylated spirit (IMS) in water (100%, 90%, 75%) and washed in distilled water. Sections were then permeabilised in 0.1% Triton X-100 for 8 min and antigen retrieval was performed by incubating sections in 10 mM sodium citrate, pH 6.0 in a 90°C waterbath for 20 min. YAP staining was then conducted using a HRP/DAB detection IHC kit (Abcam #ab64264) following manufacturer instructions. Briefly, sections were incubated in hydrogen peroxide block for 10 min, washed twice in PBS and treated with protein block for 10 min. Sections were then washed again in PBS and probed with primary antibody (1:100) (Abcam active YAP #ab205270) overnight at 4°C. The following day, sections were washed 4 times in PBS and incubated with biotinylated goat anti-polyvalent for 10 min, washed a further 4 times in PBS and treated with streptavidin peroxidase for 10 min. Sections were washed 4 times in PBS and treated with freshly prepared DAB chromogen/substrate mix for 5 min, washed 4 times in PBS and counterstained in filtered haematoxylin solution for 5 min. Sections were then differentiated with acid alcohol solution for 10 s, washed in running water for 5 min and sequentially dehydrated in increasing percentages of IMS in water (75%, 90%, 100%), cleared in histoclear for 10 min and mounted with coverslips using DPX. Sections were imaged on a 3D Histech Panoramic 250 Flash II slide scanner and 5 40x magnification images of each mouse heart sample were analysed for DAB intensity using ImageJ.

#### F4/80+ staining of histological sections

Tissue sections from level 3 of the heart were deparaffinised on a heat block at 80°C for 2 min and cleared in xylene for 1 h. Sections were then sequentially rehydrated in decreasing percentages of industrial IMS in water (100%, 90%, 75%) and washed in distilled water. Antigen retrieval was carried out by immersion of sections in 10 mM sodium citrate buffer at 95°C for 20 min followed by permeabilization with 0.3% triton X-100 in PBS for 30 min and blocking with 5% goat serum for 1 h. Sections were then probed with rabbit anti-F4/80 (1:100) and mouse anti-α-actinin primary antibodies overnight at 4°C. The following day the sections were washed and incubated with anti-mouse AlexaFluor® 647 and anti-rabbit AlexaFluor® 488 secondary antibodies (1:200) for 2 h at RT in the dark, then washed, counterstained with DAPI (1:5000), washed again and mounted under a coverslip using ProLong™ Gold Antifade Mountant. Once dried overnight, sections were imaged on a 3D Histech Panoramic 250 Flash II slide scanner.

#### Immunoprecipitation experiment

NRCM were cultured in 6 well plates at 2x10ˆ6 cells/well and treated with Ad-FLAG-5-HT_2B_ for 48 h to induce overexpression of 5HT_2B_. Cells were washed in ice-cold PBS, lysed with non-denaturing lysis buffer (50 mM Tris-HCL pH 8, 150 mM NaCl, 1% Triton X-100) containing 1X Pierce Protease Inhibitors (ThermoFisher Cat# A32963) for 20 min at 4°C and the lysates were then centrifuged at 8000 rpm at 4°C for 6 min. Protein concentration of the supernatant was measured by BCA assay as described and 2 tubes of lysate were prepared such that 1 mg total protein was contained in 500 ul lysis buffer. 50 μl Pierce™ Protein A/G Agarose bead slurry (ThermoFisher Cat#20421) was incubated with each lysate on a rotary mixer for 1 h at 4°C to pre-clear the lysates. Samples were centrifuged at 1000 rpm for 1 min at 4°C and the pre-cleared supernatants were recovered. 10 uL mouse anti-DYKDDDDK tag monoclonal antibody (Proteintech Cat No# 66008-4-Ig) was added to one tube of lysate while 10 uL mouse anti-lacZ antibody (ThermoFisher Cat# MA1-152) was added to the control tube of lysate on a rotary mixer overnight at 4°C. The following day, 50 uL Pierce™ Protein A/G Agarose bead slurry was added to each tube and incubated on a rotary mixer overnight at 4°C. The IP mixtures were then centrifuged at 1000 rpm for 1 min at 4°C and the bead pellets were washed 4 times in ice-cold TBS-T containing 1X Pierce Protease Inhibitors. The pellets were resuspended in 6X Lamelli buffer, heated at 95°C for 5 min and centrifuged at 10,000xg for 3 minutes at RT to dissociate the beads. Supernatants were then loaded onto 4–20% Mini-PROTEAN® TGX™ precast protein gels (Bio-Rad), separated by SDS-PAGE and transferred to nitrocellulose membranes as described. Membranes were blocked with 5% BSA in TBS-T for 1 h and probed overnight with rabbit anti-LATS1 (1:1000) and anti- DYKDDDDK tag monoclonal antibody at 4°C. The following day they were washed three times for 5 min with TBS-T and incubated with HRP-conjugated secondary antibody for 2 h at RT. The membranes were washed a further three times for 5 min then developed with ECL and imaged as described.

#### Determination of protein expression by western blot

##### Protein extraction

For *in-vitro* experiments, cells were seeded onto 6-well plates and treated as described above, after which they were washed in PBS and lysed with 100 μL RIPA buffer for 20 min at 4°C. The lysates were collected into tubes using a cell scraper and centrifuged at 8000 rpm for 6 min at 4°C. The supernatant containing the protein was transferred to a fresh tube and stored at -20°C. For *in-vivo* experiments, mouse hearts were collected, snap frozen in liquid nitrogen and stored at −80°C. Approximately half the ventricle tissue from each mouse heart was homogenised in 500 μL RIPA buffer using a Dounce homogeniser and samples were lysed for 20 min at 4°C then centrifuged at 8000 rpm for 6 min at 4°C. The supernatant containing the protein was transferred to a fresh tube and stored at -20°C.

##### Quantification of protein concentration

Protein concentration was quantified using a bicinchoninic acid (BCA) assay kit (Pierce™) following manufacturer instructions. Briefly, an 8-point standard curve was prepared by serial dilution of kit BSA standard, and protein samples were diluted in RIPA buffer (1:5 for cell-derived protein, 1:10 for tissue-derived protein). 25 μL standards and unknown samples were loaded onto a 96-well plate and 200 μL BCA working reagent was added to the wells. The BCA working reagent contains copper sulphate, and when the copper ions are exposed to protein in the presence of an alkaline medium, they are reduced from Cu^2+^ to Cu^+^, and the free Cu^+^ ions react with the BCA resulting in a purple-coloured product. After 30 min incubation at 37°C, the absorbance at 562 nm was measured using a Multiskan ascent spectrophotometer. The absorbance values of the standards were plotted against their known log_10_ concentrations, and a standard curve was fitted with a 4-parameter logistic regression equation. Sample protein concentrations were derived from the antilog of their x values interpolated from the standard curve and multiplied by 5 (cell samples) or 10 (tissue samples).

##### Gel electrophoresis and membrane transfer

Protein samples were denatured with 2x Laemmli buffer (Bio-Rad) and heated to 95°C for 5 min. 20 μg denatured protein samples were loaded into 4–20% Mini-PROTEAN® TGX™ precast protein gels (Bio-Rad) and separated by SDS-polyacrylamide gel electrophoresis (SDS-PAGE) and run alongside Precision Plus Protein™ dual colour standard (Bio-Rad) in a tank containing Tris-glycine running buffer (25 mM Tris base, 0.25 M glycine, 0.1% SDS) at 120 V for 60 min. Proteins were then transferred to nitrocellulose membranes using a Trans-Blot Turbo Transfer System (Bio-Rad) at 2.5 A/25V for 7 min.

##### Antibody probing

Membranes were blocked with 5% BSA in Tris-Buffered Saline (TBS) containing 0.05% Tween 20 (TBS-T) for 1 h. The membranes were then probed primary antibody overnight at 4°C and the following day they were washed three times for 5 min with TBS-T and incubated with HRP-conjugated secondary antibody for 2 h at RT. The membranes were washed a further three times for 5 min then developed with enhanced chemiluminescence (ECL) western blotting reagents (GE Healthcare) and images of the blots were acquired using a ChemiDoc XRS+ with Image Lab 3.0 software (Bio-Rad). The membranes were washed, reprobed with HRP-conjugated primary housekeeper antibody for 1 h at RT and imaged as described in this section.

### Quantification and statistical analysis

All data are reported as mean ± standard error of the mean (SEM) and, depending on experimental design, data were normalised to a relevant control with specific details given explicitly in figure legends. Statistical analyses were performed using GraphPad Prism 10 (GraphPad Software, LLC). We used the Shapiro-Wilk test to analyse the normal distribution of the data. Comparisons between two experimental groups were conducted using independent t-tests if the data was normally distributed or Mann-Whitney U test if the data was not normally distributed. If there were more than two experimental groups, we used one-way analysis of variance (ANOVA) with Tukey’s multiple comparisons if the data was normally distributed, or Kruskal-Wallis test if the data was not normally distributed. For analysis of survival data, a Log rank Mantel-Cox test was performed. In all cases a p value ≤ 0.05 was considered to be significant. Information on statistical tests used, n number, and P values from each experiment are presented in the figures and figure legends.
