# Supplementary material for: The serotonin receptor 2b (5-HT2B) modulates heart remodeling following myocardial infarction via regulation of Hippo pathway
Source: iScience. 2026 Jan 27;29(2):114825. doi: 10.1016/j.isci.2026.114825 (PMC12915196; doi:10.1016/j.isci.2026.114825)
Supplement: Document S1. Figures S1–S7 and Tables S1–S3 [file mmc1.pdf]

iScience, Volume *29*

Supplemental information

The serotonin receptor 2b (5-HT_{2B}) modulates heart remodeling following myocardial infarction via regulation of Hippo pathway

Ryan Potter, Min Zi, Sukhpal Prehar, Tara McNulty, Dowan Kwon, Ellie England, Maram Almutairi, Nicholas Stafford, Ardiansah Bayu Nugroho, Efta Triastuti, Luc Maroteaux, Elizabeth J. Cartwright, and Delvac Oceandy

SUPPLEMENTARY INFORMATION

The serotonin receptor 2b (5-HT_{2B}) modulates left ventricular remodelling following myocardial infarction through regulation of Hippo pathway

Ryan Potter[1], Min Zi[1], Sukhpal Prehar[1], Tara McNulty[1], Dowan Kwon[1], Ellie England[1], Maram Almutairi[1], Nicholas Stafford[2], Ardiansah Bayu Nugroho[1], Efta Triastuti[1], Luc Maroteaux[3], Elizabeth J Cartwright[1], Delvac Oceandy[1,*].

[1]Division of Cardiovascular Sciences, Faculty of Biology, Medicine and Health, The University of Manchester, Manchester Academic Health Science Centre, Manchester, United Kingdom.

[2]Division of Diabetes, Endocrinology and Gastroenterology, Faculty of Biology, Medicine and Health, The University of Manchester, Manchester Academic Health Science Centre, Manchester, United Kingdom

[3]Institut du Fer à Moulin, U1270 INSERM, Sorbonne Université, 17 rue du Fer à Moulin, 75005 Paris, France

Tables S1-S3.

Figures S1-S7.

Table S1. In-Vitro Transcription Reaction Reagents.

Reagent	Stock Concentration	Volume Per Reaction	Final Concentration
ATP	75 mM	2 μL	7.5 mM
CTP	75 mM	2 μL	7.5 mM
GTP	75 mM	0.4 μL	1.5 mM
N1-methyl-pseudoUTP*	75 mM	2 μL	7.5 mM
Anti Reverse Cap Analog, 3'-O-Me-m7G(5')ppp(5')G (ARCA)+	60 mM	2 μL	6 mM
10X T7 Reaction Buffer	NA	2 μL	NA
T7 RNA Polymerase	NA	2 μL	NA
Nuclease-free water	NA	6.6 μL	NA
Linearised Template DNA	1 μg/μL	1 μL	NA

Abbreviations: ATP = adenosine triphosphate, CTP = cytidine triphosphate, GTP = guanosine triphosphate, UTP = uridine triphosphate, NA = not applicable.

*Jena Bioscience #NU890-L

+Stratech #B8175-APE

Table S2. PolyA Tailing Reaction Reagents.

Reagent	Volume Per Reaction (µL)
MEGAscript® T7 IVT Reaction RNA	20
Nuclease-free water	35
5X E-PAP Buffer	20
25 mM $MnCl_2$	10
10 mM ATP	10
E-PAP Enzyme	4*

Abbreviations: IVT = in-vitro transcription, E-PAP = *E. coli* Poly(A) Polymerase I, ATP = adenosine triphosphate.

Table S3. Formulae used to calculate cardiac structural and functional parameters.

Parameter	Formula
Left Ventricle Mass	1.055 x [dD + dPW + dIVS)^3 – dLVD^3]
Ejection Fraction	[(dVol-sVol)/dVol] x 100

Abbreviations: *d* = diastolic, *s* = systolic, D = diameter, PW = posterior wall, IVS = interventricular septum, LV = left ventricle, Vol = volume.

NB: dVol = (1.047 x dD)^3; sVol = (1.047 x sD)^3

Figure S1

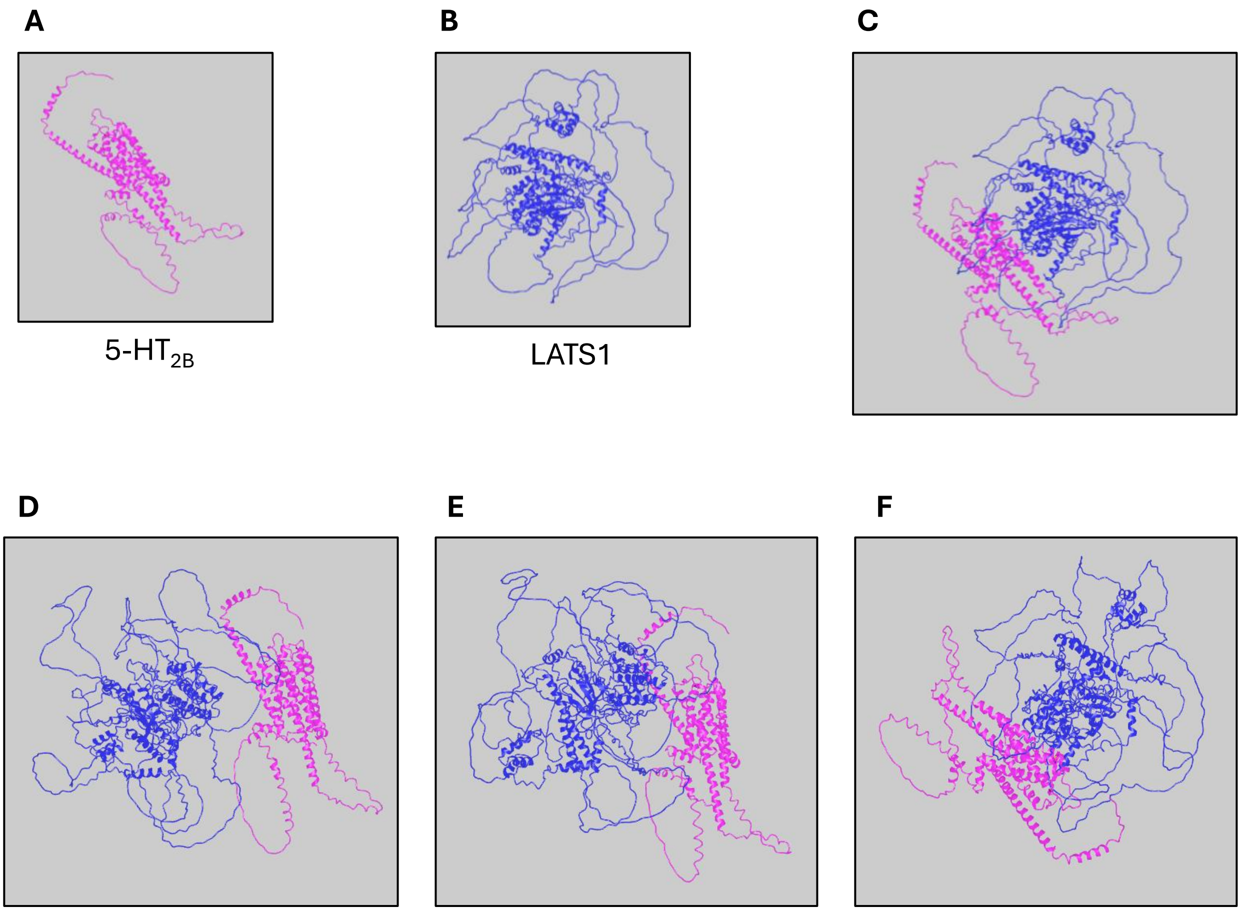

Figure S1. Protein docking model of mouse 5-HT$_{2B}$ – LATS1
The 3D structure of **A)** mouse 5-HT$_{2B}$ and **B**) mouse LATS1 were predicted using AlphaFold[1-2]. The resulting PDB files were used to predict how these proteins might interact to form a complex by using docking algorithm (LZerD protein docking tool)[3-4.] The four docking models with highest scores based on GOAP[5], DFIRE[6] and ITScore[7] systems are depicted in panels **C-F**.

Figure S2

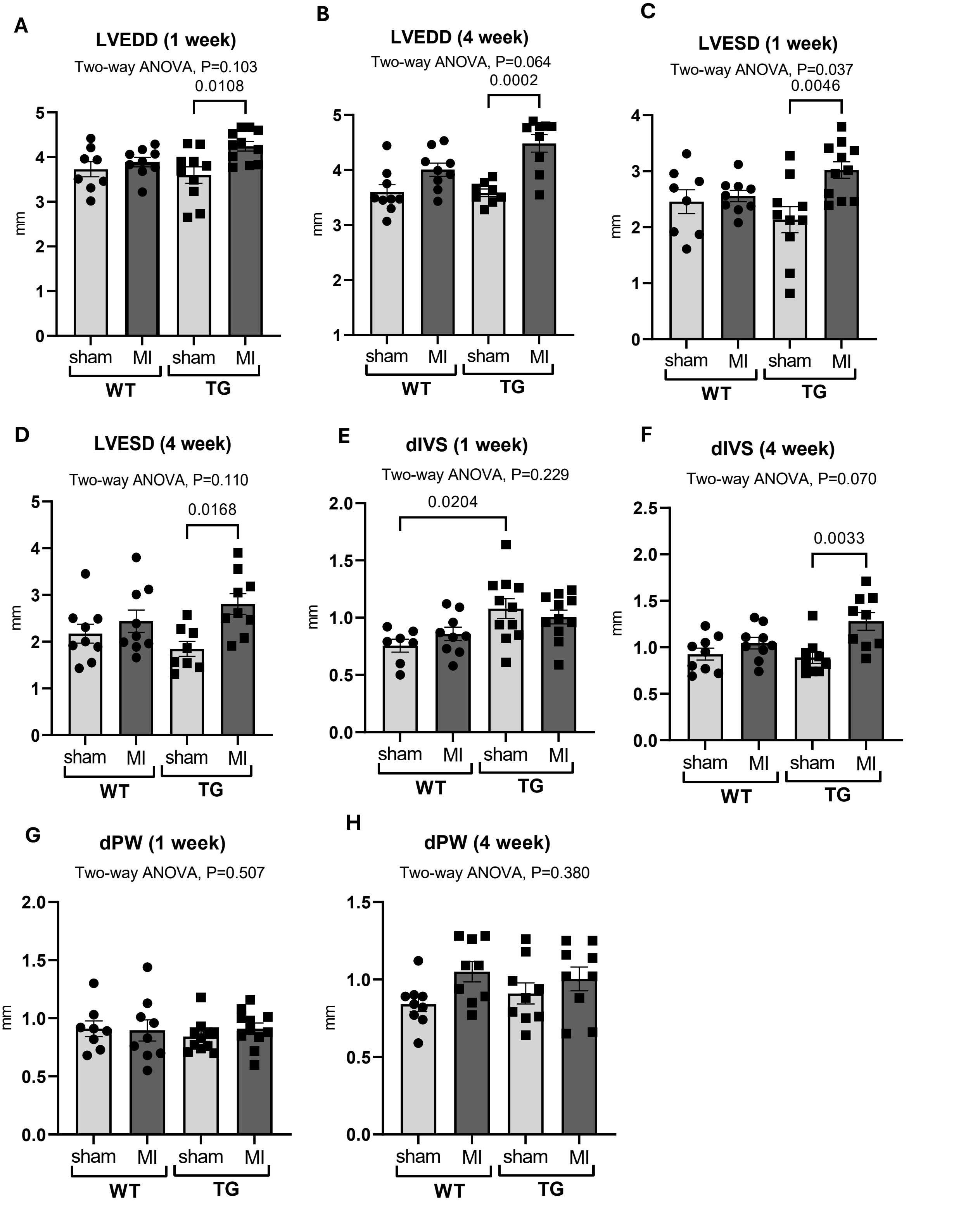

Figure S2. Echocardiography analysis of 5-HT$_{2B}$ cTG mice at 4 weeks after MI (**A**) LV end-diastolic diameter was increased in the transgenic MI group at 1-week post-MI and (**B**) 4-weeks post-MI. (**C**) LV end-systolic diameter was also increased in the transgenic MI group at 1-week post-MI and (**D**) 4 weeks-post-MI. (**E**) Diastolic interventricular septum thickness was not significantly different at 1-week post-MI, although by (**F**) 4 weeks post-MI diastolic IVS thickness was elevated in the TG MI group. (**G**) No changes in diastolic posterior wall thickness were detected at either 1-week post-MI, or (**H**) by 4 weeks post-MI. (n=8-11 in each group for all analyses above). Data are presented as mean $\pm$ SEM. Statistical test: Two-way ANOVA followed by posthoc multiple comparisons

Figure S3

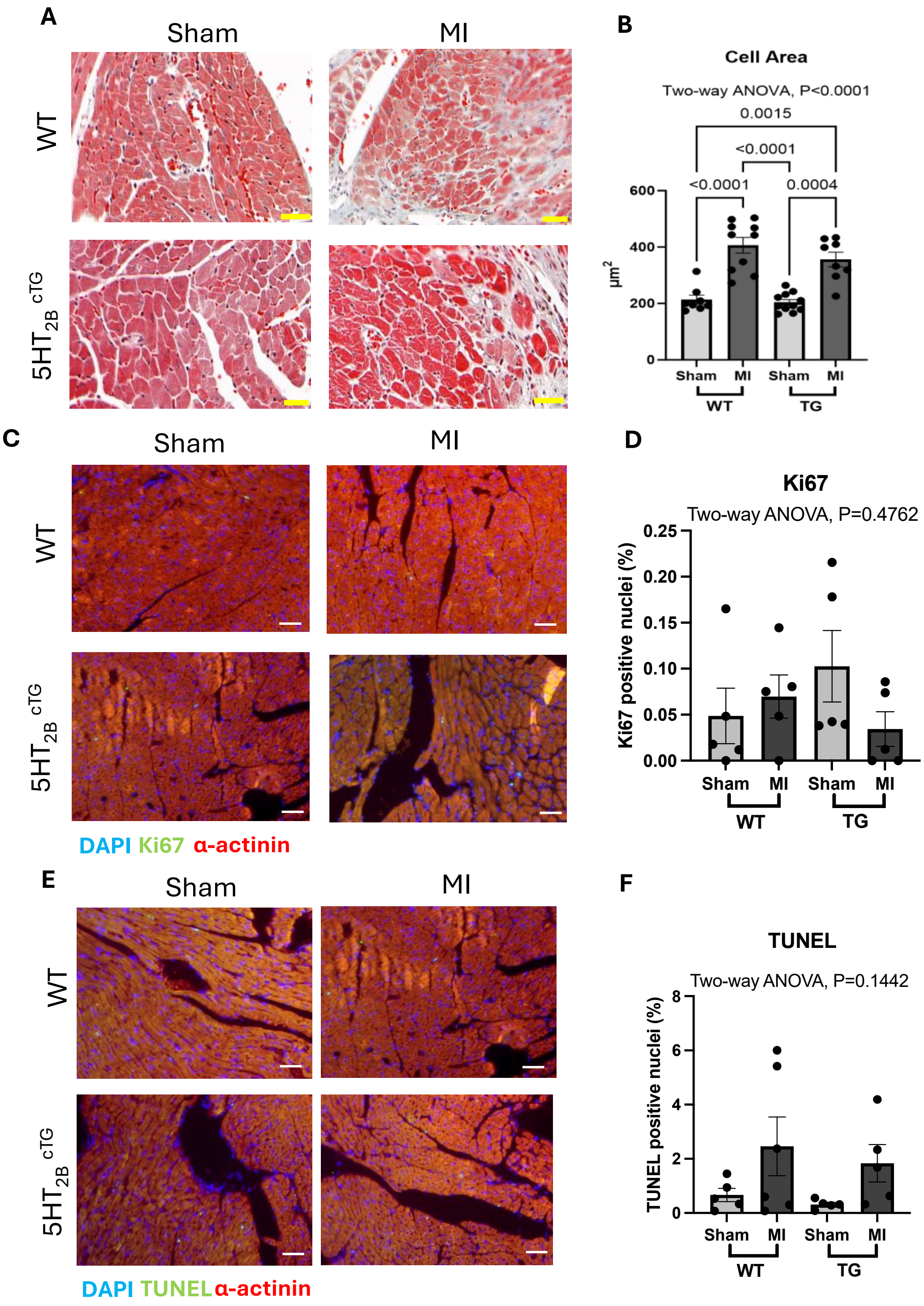

Figure S3. Transgenic Overexpression of 5-HT$_{2B}$ did not affect Cell Size, Proliferation or Apoptosis. (**A**) Representative magnified Masson's Trichrome-stained heart sections (scale bars=40 μm) and (**B**) quantification of cell size indicated that relative to sham mice cell size appeared to increase similarly in 5-HT$_{2B}^{cTG}$ (TG) and WT mice (n=7-10 in each group) (**C**) Representative immunofluorescence images of Ki67-stained heart sections (scale bars=100 μm) and (**D**) quantification of Ki67 detection showed no significant increase in cell proliferation (n=5 in each group). (**E**) Representative immunofluorescence images detecting TUNEL-labelled apoptotic nuclei and (**F**) quantification of TUNEL-positive cardiomyocyte nuclei suggests no significant differences in apoptosis between groups (n=5 in each group). Data are presented as mean ± SEM. Statistical test used: Two-way ANOVA followed by posthoc multiple comparisons.

Figure S4

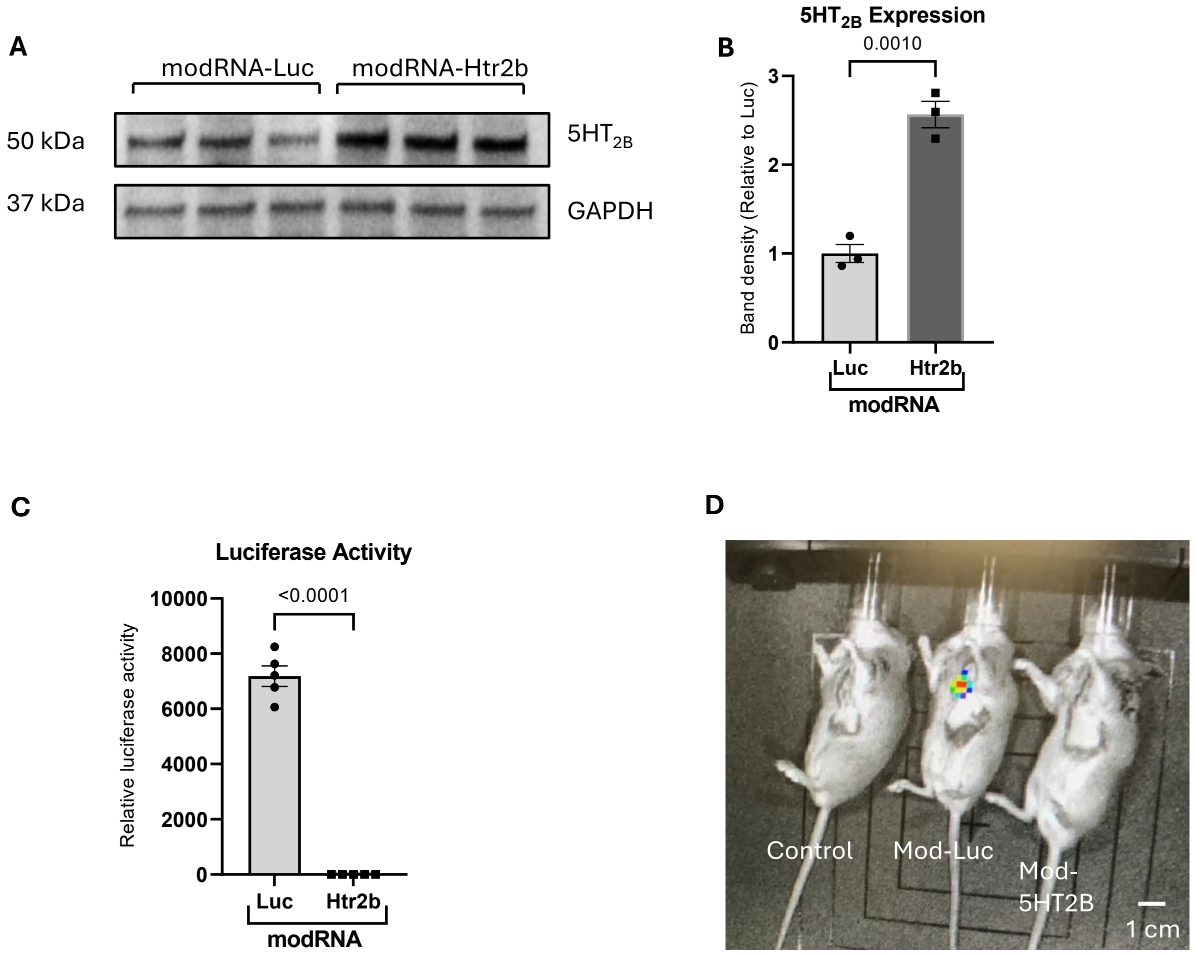

Figure S4. Validation of the modRNA-mediated overexpression model. (**A**) Western blot analysis of protein lysates from H9C2 cells transfected with modRNA and (**B**) quantification of $5\text{-}HT_{2B}$ overexpression (n=3 independent experiments). (**C**) Luciferase activity in H9C2 cells was potently induced by modRNA-Luc but not by modRNA-Htr2b (n=5 independent experiments). Data are presented as mean $\pm$ SEM. Statistical test used: Student's t-test.(**D**) Intracardiac injection of 50 µg modRNA-Luc produced a strong bioluminescent signal localised to the mouse heart at 48 h upon intraperitoneal injection of VivoGlo substrate that was absent in the modRNA-Htr2b mouse.

Figure S5

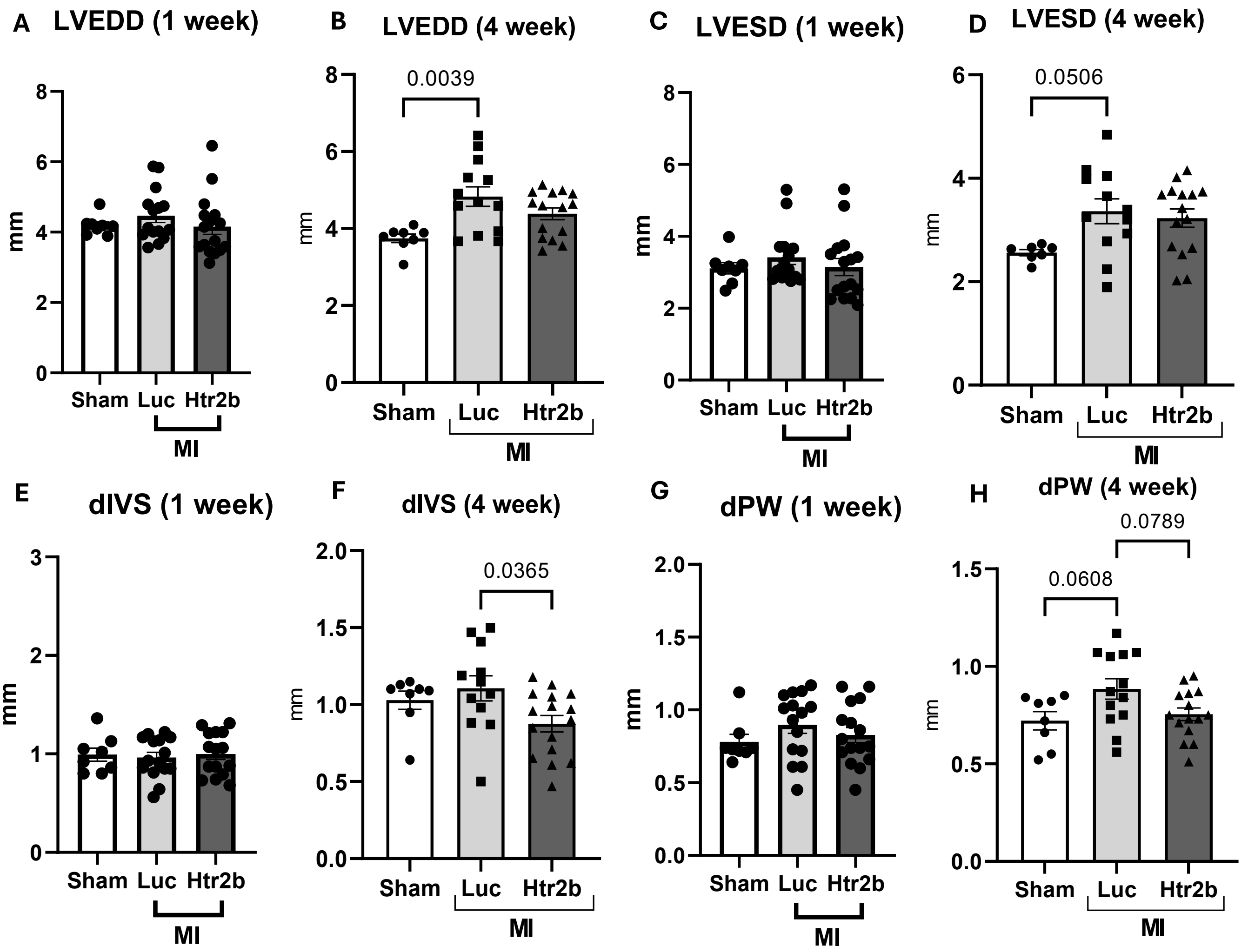

Figure S5. Echocardiography analysis of mice with modRNA-mediated 5-HT$_{2B}$ overexpression following MI. (**A**) LV end-diastolic diameter (LVEDD) was unchanged at 1-week post-MI however by (**B**) 4-weeks post-MI, LVEDD was significantly higher in the modRNA-Luc control mice but not in the modRNA-Htr2b mice. (**C**) LV end-systolic diameter (LVESD) was also unchanged at 1-week post-MI but by (**D**) 4 weeks-post-MI, LVESD trended highest in the modRNA-Luc group. (**E**) Diastolic interventricular septum thickness (dIVS) was not significantly different at 1-week post-MI, although by (**F**) 4 weeks post-MI diastolic IVS thickness was significantly higher in the modRNA-Luc group compared to the modRNA-Htr2b group. (**G**) No changes in diastolic posterior wall thickness (dPW) were detected at either 1-week post-MI, or (**H**) by 4 weeks post-MI, however PW thickness trended highest in the modRNA-Luc group. (n=8-16 in each group for all analyses above). Data are presented as mean ± SEM. Statistical test used: One-way ANOVA followed by posthoc multiple comparisons.

Figure S6

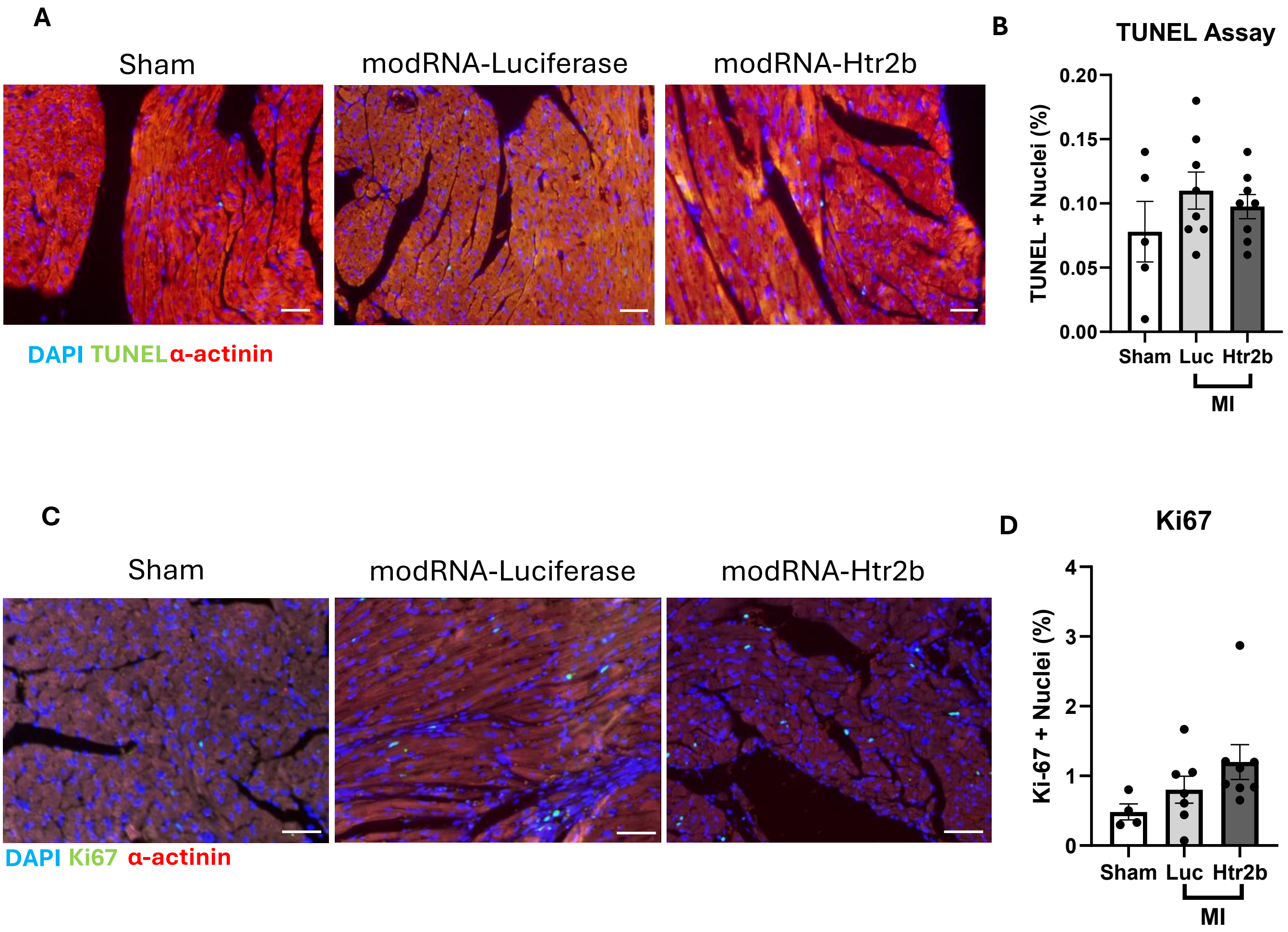

Figure S6. (**A**) Representative immunofluorescence images detecting TUNEL-labelled apoptotic nuclei (scale bars= 100 μm) and (**B**) the quantification of TUNEL-positive cardiomyocyte nuclei suggests no differences in apoptosis between mice treated with modRNA-luciferase and modRNA-Htr2b after MI. (**C**) Representative immunofluorescence images of Ki67-stained heart section (scale bars=100 μm) and (**D**) quantification of Ki67 detection showed no significant increase in proliferation although detection trended highest in the modRNA-Htr2b group. (n=5-8 in each group). Data are presented as mean ± SEM. Statistical test used: One-way ANOVA followed by posthoc multiple comparisons.

Figure S7

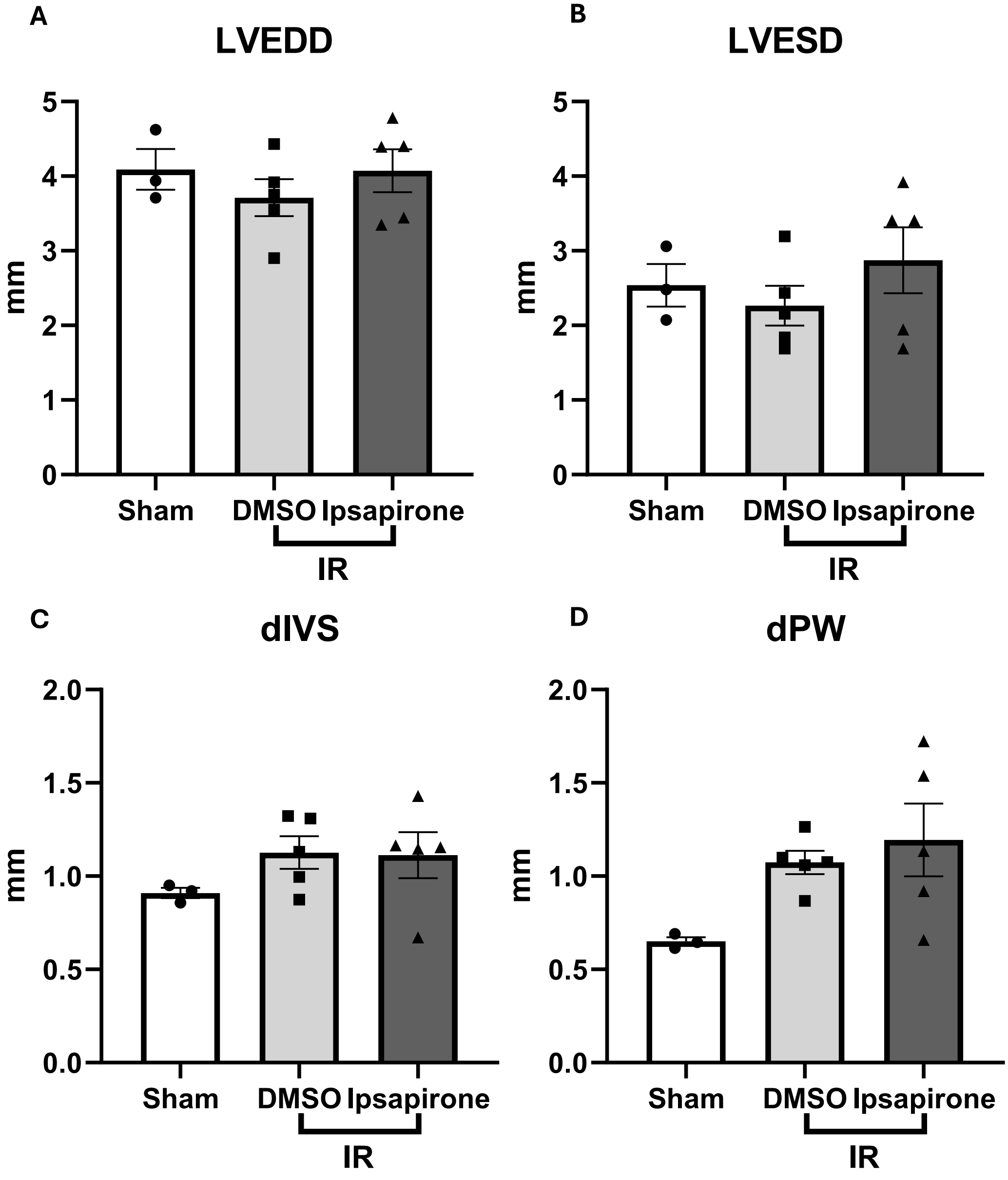

Figure S7. (**A**) LV end-diastolic diameter (LVEDD) and (**B**) LV end-systolic diameter(LVESD) were not significantly altered by 2-weeks post-IR though trended highest in the ipsapirone groups. (**C**) Diastolic interventricular septum thickness (dIVS) was not significantly different at 2-weeks post-IR and (**D**) No changes in diastolic posterior wall thickness (dPW) were detected by 2 weeks post-IR. (Sham, n=4; MI+DMSO, n=5; MI+Ipsapirone, n=5). Data are presented as mean $\pm$ SEM. Statistical test used:One-way ANOVA followed by posthoc multiple comparisons.

References

1. Jumper, J., Evans, R., Pritzel, A., Green, T., Figurnov, M., Ronneberger, O., Tunyasuvunakool, K., Bates, R., Zidek, A., Potapenko, A., et al. (2021). Highly accurate protein structure prediction with AlphaFold. Nature *596*, 583-589. 10.1038/s41586-021-03819-2.
2. Fleming, J., Magana, P., Nair, S., Tsenkov, M., Bertoni, D., Pidruchna, I., Lima Afonso, M.Q., Midlik, A., Paramval, U., Zidek, A., et al. (2025). AlphaFold Protein Structure Database and 3D-Beacons: New Data and Capabilities. J Mol Biol *437*, 168967. 10.1016/j.jmb.2025.168967.
3. Esquivel-Rodriguez, J., Filos-Gonzalez, V., Li, B., and Kihara, D. (2014). Pairwise and multimeric protein-protein docking using the LZerD program suite. Methods Mol Biol *1137*, 209-234. 10.1007/978-1-4939-0366-5_15.
4. Christoffer, C., Chen, S., Bharadwaj, V., Aderinwale, T., Kumar, V., Hormati, M., and Kihara, D. (2021). LZerD webserver for pairwise and multiple protein-protein docking. Nucleic Acids Res *49*, W359-W365. 10.1093/nar/gkab336.
5. Zhou, H., and Skolnick, J. (2011). GOAP: a generalized orientation-dependent, all-atom statistical potential for protein structure prediction. Biophys J *101*, 2043-2052. 10.1016/j.bpj.2011.09.012.
6. Zhou, H., and Zhou, Y. (2002). Distance-scaled, finite ideal-gas reference state improves structure-derived potentials of mean force for structure selection and stability prediction. Protein Sci *11*, 2714-2726. 10.1110/ps.0217002.
7. Huang, S.Y., and Zou, X. (2011). Statistical mechanics-based method to extract atomic distance-dependent potentials from protein structures. Proteins *79*, 2648-2661. 10.1002/prot.23086.
